# Complex I and II Subunit Gene Duplications Provide Increased Fitness to Worms

**DOI:** 10.3389/fgene.2019.01043

**Published:** 2019-10-25

**Authors:** Lucía Otero, Cecilia Martínez-Rosales, Exequiel Barrera, Sergio Pantano, Gustavo Salinas

**Affiliations:** ^1^Laboratorio de Biología de Gusanos, Unidad Mixta Departamento de Biociencias, Facultad de Química, Universidad de la República–Institut Pasteur de Montevideo, Montevideo, Uruguay; ^2^Laboratorio de Simulaciones Biomoleculares, Institut Pasteur de Montevideo, Montevideo, Uruguay

**Keywords:** rhodoquinone, *C. elegans*, nematode, platyhelminth, electron transport chain, hypoxia, helminth, gas-1

## Abstract

Helminths use an alternative mitochondrial electron transport chain (ETC) under hypoxic conditions, such as those found in the gastrointestinal tract. In this alternative ETC, fumarate is the final electron acceptor and rhodoquinone (RQ) serves as an electron carrier. RQ receives electrons from reduced nicotinamide adenine dinucleotide through complex I and donates electrons to fumarate through complex II. In this latter reaction, complex II functions in the opposite direction to the conventional ETC (i.e., as fumarate reductase instead of succinate dehydrogenase). Studies in *Ascaris suum* indicate that this is possible due to changes in complex II, involving alternative succinate dehydrogenase (SDH) subunits SDHA and SDHD, derived from duplicated genes. We analyzed helminth genomes and found that distinct lineages have different gene duplications of complex II subunits (SDHA, SDHB, SDHC, and SDHD). Similarly, we found lineage-specific duplications in genes encoding complex I subunits that interact with quinones (NDUF2 and NDUF7). The phylogenetic analysis of ETC subunits revealed a complex history with independent evolutionary events involving gene duplications and losses. Our results indicated that there is not a common evolutionary event related to ETC subunit genes linked to RQ. The free-living nematode *Caenorhabditis elegans* uses RQ and has two genes encoding SDHA (*sdha-1* and *sdha-2*) and two genes encoding NDUF2 (*nduf2-1* and *nduf2-2*). *sdha-1* and *nduf2-1* are essential genes and have a similar expression pattern during *C. elegans* lifecycle. Using knockout strains, we found that *sdha-2* and *nduf2-2* are not essential, even in hypoxia. Yet, *sdha-2* and *nduf2-2* expression is increased in the early embryo and in dauer larvae, stages where there is low oxygen tension. Strikingly, *sdha-1* and *sdha-2* as well as *nduf2-1* and *nduf2-2* showed inverted expression profiles during the *C. elegans* life cycle. Finally, we found that *sdha-2* and *nduf2-2* knockout mutant strain progeny is affected. Our results indicate that different complex I and II subunit gene duplications provide increased fitness to worms.

## Introduction

**Figure 1:**
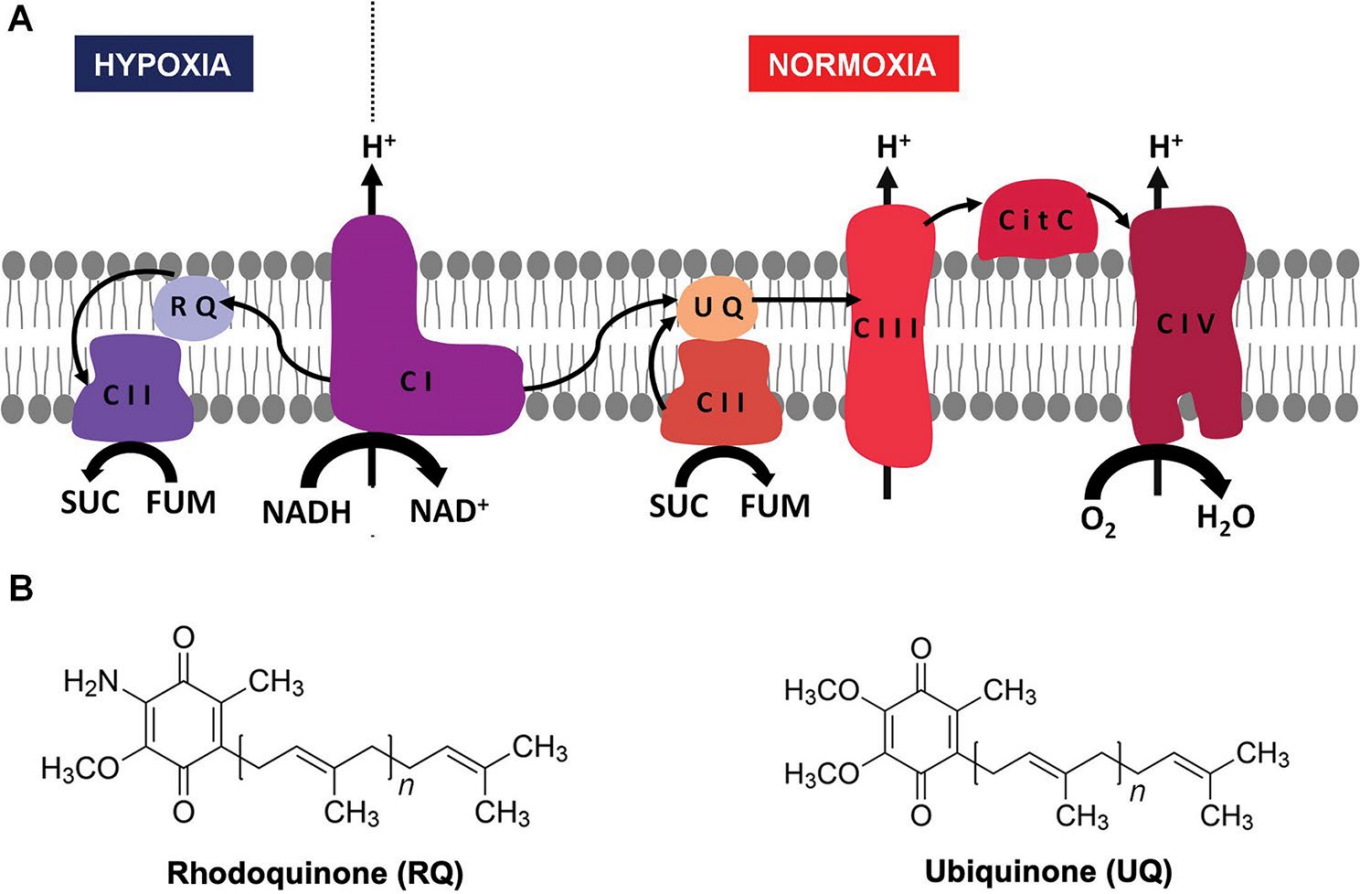
Helminth mitochondrial electron transport chains in normoxia and hypoxia. In the presence of oxygen, electrons from reduced nicotinamide adenine dinucleotide (NADH) and succinate are transferred to UQ through complex I and II, then from UQ to cytochrome c through complex III, and finally from cytochrome c to O_2_ through complex IV (**A**, right). When there is no oxygen available (**A**, left) the electron transport chain functions with only two complexes. Electrons are transferred from NADH to RQ through complex I and then from RQ to fumarate through complex II. In both cases, a proton gradient across the inner membrane is generated, which is used to produce ATP through complex V (not shown). The structures of UQ (right) and RQ (left) are shown in **(B)**.

Energy is essential to all forms of life. Helminths live part of their life cycle under hypoxic conditions and have an energy metabolism different from their hosts. Under hypoxia, helminths use fumarate instead of oxygen as a final electron acceptor of the electron transport chain (ETC) (**Figure 1A**) (Hellemond et al., 2003; Harada et al., 2013). This is achieved by means of an alternative ETC, in which rhodoquinone (RQ) serves as an electron carrier (Van Hellemond et al., 1995; Hellemond et al., 2003;Harada et al., 2013). RQ is a redox-active quinone structurally similar to the conventional ubiquinone (UQ), differing only in the 6-methoxy substituent of UQ that is changed to an amino substituent in RQ (**Figure 1B**) (Moore and Folkers, 1965). This change confers RQ a lower redox potential than UQ (-63 and 43 mV, respectively) (Erabi et al., 1975). This allows RQ to receive electrons from reduced nicotinamide adenine dinucleotide (NADH) through complex I and to reduce fumarate to succinate (redox potential 30 mV) through complex II. Thus, in this alternative ETC, complex II functions as a fumarate reductase (FRD), in the opposite direction to the conventional ETC in which complex II functions as succinate dehydrogenase (SDH) (Van Hellemond et al., 1995)(Tielens and Van Hellemond, 1998). SDH oxidizes succinate to fumarate and reduces UQ. Importantly, the alternative ETC also allows proton pumping through complex I. The resulting proton gradient is coupled to ATP synthesis by mitochondrial ATP synthase. The fumarate and NADH used in this ETC are generated in the malate dismutation pathway (Tielens and Van Hellemond, 1998). This pathway yields between 5 and 6 mol of ATP per mol of glucose. A similar ETC that uses RQ is also present in some prokaryotes, protists, and other animals that alternate cycles of normoxia and hypoxia, such as bivalves and freshwater snails (Van Hellemond et al., 1995).

RQ has been found in all helminths where its presence has been examined, being more abundant in stages that dwell in hypoxic environments (Sato and Ozawa, 1969; Allen, 1973; Van Hellemond et al., 1995). Importantly, RQ has also been associated with hypoxia in the free-living nematode *Caenorhabditis elegans* (Takamiya et al., 1999), an amenable model for genetic studies. It is thought that the use RQ as an electron carrier requires adjustments in ETC complex II subunits. Several studies in *Ascaris suum*, a nematode in which biochemical studies are feasible, have shown that the use of RQ is possible due to changes in complex II subunits (Takamiya et al., 1993; Kuramochi et al., 1994; Iwata et al., 2008; Amino et al., 2003). Mitochondria from aerobic developmental stages of *A. suum*, larval stages L2 and L3, contain mainly UQ. In contrast, mitochondria from adult worms, which live in an oxygen-deprived environment, contain only RQ (Takamiya et al., 1993). The transition from normoxia to hypoxia is accompanied by an exchange of the SDHA and SDHD subunits of complex II, leading to the change in enzymatic activities of complex II, from SDH-UQ reductase to FRD-rhodoquinol oxidase (Takamiya et al., 1993; Saruta et al., 1995; Amino et al., 2003; Iwata et al., 2008). In the nematode *Haemonchus contortus*, two genes encoding distinct SDHB subunits were identified and showed different expression patterns during development (Roos and Tielens, 1994). This phenomenon has also been linked to a metabolic transition due to a change in oxygen tension. In addition, FRD and SDH activities have been detected in other helminths (Fioravanti et al., 1998; Matsumoto et al., 2008), but the complex II composition has not been examined. In contrast to complex II, no transitions associated with oxygen tension have been reported in helminth complex I (NADH dehydrogenase). The genes encoding ETC complexes I and II have not been studied in detail after helminth genomes have been made publicly available.

We examined nematode and platyhelminth genomes and found diverse gene duplications in complex II subunits. Furthermore, gene duplications in complex I subunits that interact with quinones were identified in helminth genomes. Using *C. elegans*, we studied the role of complex I and II subunits duplications.

## Results

### Different Helminths Have Dissimilar Complex I and II Subunit Duplications

**Figure 2:**
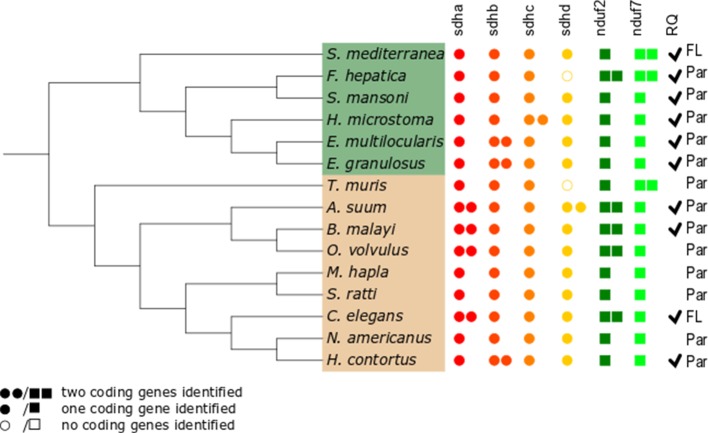
Distribution of genes encoding electron transport chain complex II subunits and quinone-binding complex I subunits in nematodes and platyhelminths. The nematode (brown) and platyhelminth (green) species analyzed represent different clades or lineages and are shown according to their phylogenetic relationships. The NCBI taxonomy was used and represented using the Interactive Tree of Life online tool itol.embl.de (Letunic and Bork, 2019). Presence or absence of genes corresponding to all complex II subunits, *sdha*, *sdhb*, *sdhc*, and *sdhd*, and to complex I *nduf2* and *nduf7* genes was analyzed. Filled circles represent complex II genes; filled squares represent complex I genes. Two filled circles or squares represent presence of two coding genes identified for the subunit. Open circles indicate that no coding gene was identified. Par and FL denotes parasitic and free-living species, respectively. Ticks indicate the reported presence of RQ (Allen, 1973; Fioravanti and Kim, 1988; Van Hellemond et al., 1995; Van Hellemond, 1997;Takamiya et al., 1999; Shiobara et al., 2015). The absence of tick denotes species in which RQ presence has not been examined.

Since gene duplications of complex II subunits have been described in some helminths, we examined the genes encoding complex II in representative and well-annotated parasitic and free-living nematode and platyhelminth genomes. Interestingly, we observed diversity in complex II genes. The absence/presence of complex II genes is presented in **Figure 2** (alignments for all subunits studied are available in **Supplementary Figure 1**).

**Figure 3:**
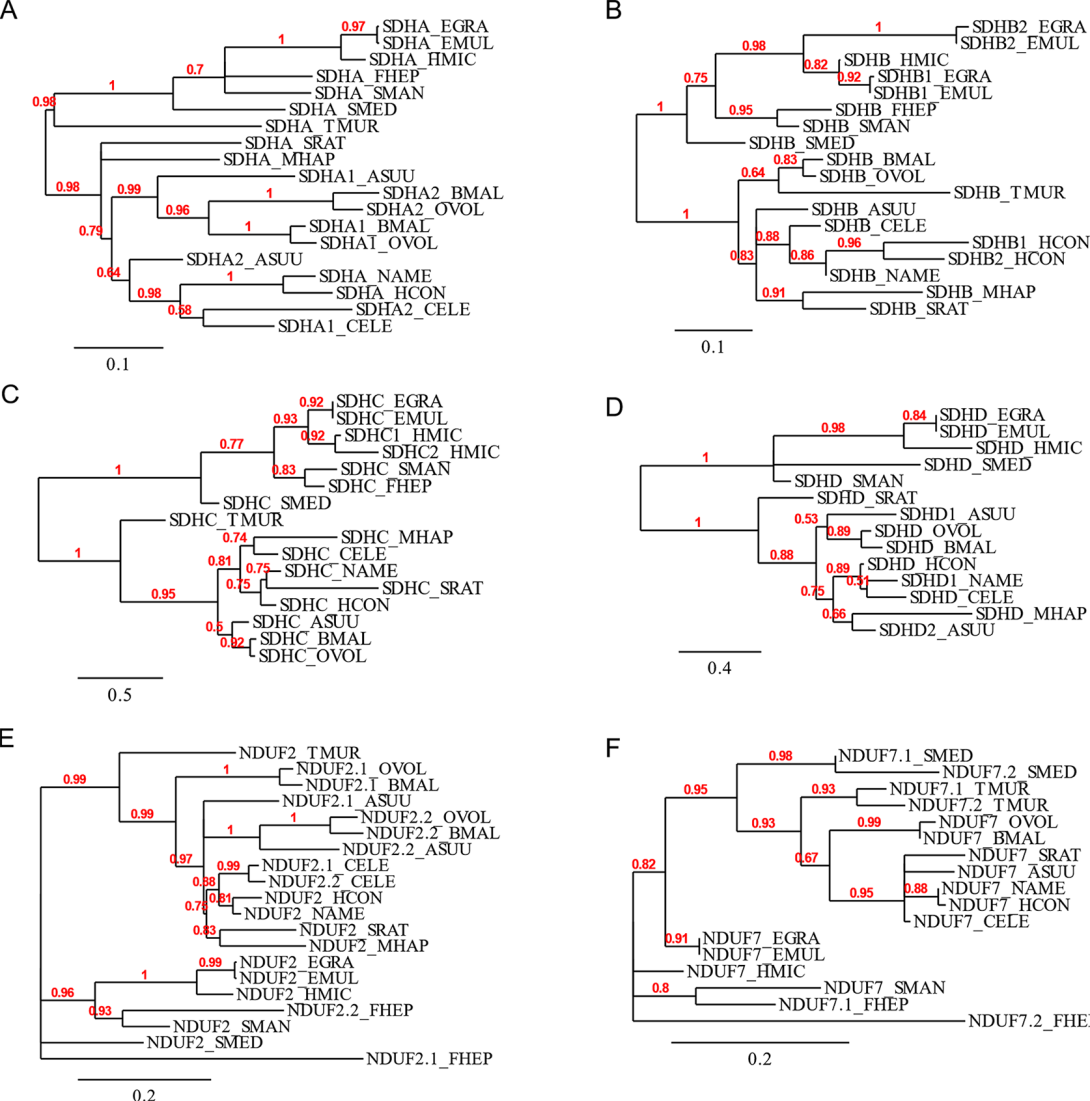
Phylogenetic trees of complex II and nuclear-encoded quinone-binding complex I subunits. Phylograms of **(A)** SDHA, **(B)** SDHB, **(C)** SDHC, **(D)** SDHD, **(E)** NDUF2, and **(F)** NDUF7. Multiple sequence alignments were performed with MUSCLE 3.8.31 and curated with Gblocks 0.91b. Phylograms were inferred using PhyML 3.0 from http://www.phylogeny.fr. The values above each branch indicate bootstrap values. Polytomies denote nodes for which the relationships cannot be resolved to dichotomies. Alignments are shown in **Supplementary Figure 1**.

The flavoprotein subunit (SDHA) gene was found to be duplicated in some, but not all, nematodes, whereas this gene duplication is not observed in any platyhelminth lineage (**Figure 2**). The phylogenetic analysis does not indicate a clear evolutionary event associated with *sdha* history (**Figure 3A**) and suggests that independent gene duplications have occurred in filarial and *C. elegans* lineages. Indeed, filarial SDHA-1 and SDHA-2 form different nodes, and they cluster together in a putative ancestral filarial SDHA node. Likewise, *C. elegans* SDHA-1 and SDHA-2 form a node, indicating a recent gene duplication event in this lineage. However, in the case of *A. suum*, the two SDHAs do not group together, suggesting that the two SDHA subunits diverged extensively after duplication. Alternatively, it may represent an ancient gene duplication that was subsequently lost in other lineages. This latter scenario is less parsimonious since it would imply several gene losses and subsequent duplications.

Two genes encoding alternative iron–sulfur subunits (SDHB) have been previously described in *H. contortus* (Roos and Tielens, 1994). This gene duplication is absent in all other nematode genomes analyzed. Interestingly, we found that *Echinococcus* spp. also exhibit an *sdhb* duplication in the platyhelminth lineage. The phylogenetic analysis of SDHB suggests independent gene duplications in these lineages.

Regarding the heme-containing membrane subunits (SDHC and SDHD), the results were unexpected. *sdhc* was found to be duplicated only in *Hymenolepis microstoma*, whereas *sdhd* was found to be duplicated only in *A. suum*. Strikingly, *Fasciola hepatica* and *Trichuris muris* lost *sdhd*, suggesting independent gene losses in these lineages. It is important to note that transcripts corresponding to *sdhd* were absent in these species’ transcriptomes. Since ETC subunit genes are highly expressed, the absence of *sdhd* transcripts favors the hypothesis that this gene is absent in these genomes.

Since adjustments in complex II are thought to be related to the use of alternative quinones (UQ or RQ), we also analyzed complex I subunits NDUF2 and NDUF7, involved in quinone binding. Interestingly, we found that the same nematodes that have two *sdha* genes have also two *nduf2* genes. In platyhelminths, only *F. hepatica* has two *nduf2* genes. Phylogenetic analysis of NDUF2 in nematodes suggests independent events of gene duplication in *C. elegans* and in clade III lineages. In the case of platyhelminth NDUF2, it is likely that a gene duplication event occurred in the *F. hepatica* lineage and rapidly diverged. However, an ancestral gene duplication (e.g., at the root of trematodes) followed by gene loss in independent lineages cannot be ruled out.

We also found that *T. muris*, *Schimdtea mediterranea*, and *F. hepatica* have two *nduf7*. According to the phylogenetic tree, the gene duplications observed in *T. muris* and *S. mediterranea* lineages appear to be recent events. Thus, the most likely explanation is that these gene duplication events occurred independently.

The analysis of the ETC subunit genes revealed a complex history with independent evolutionary events without an evident pattern. In the case of *A. suum*, the different complex II subunits used in the larval and adult stages have been associated with oxygen tension changes found during its lifecycle (normoxia and hypoxia, respectively) and the use of alternative quinones (UQ and RQ, respectively). However, a conclusion supported by our study is the absence of a common evolutionary event related to ETC subunit genes directly linked to RQ, the key metabolite during hypoxia.

### The Quinone-Binding Site in Normoxia and Hypoxia Complex II Is Highly Conserved

**Figure 4:**
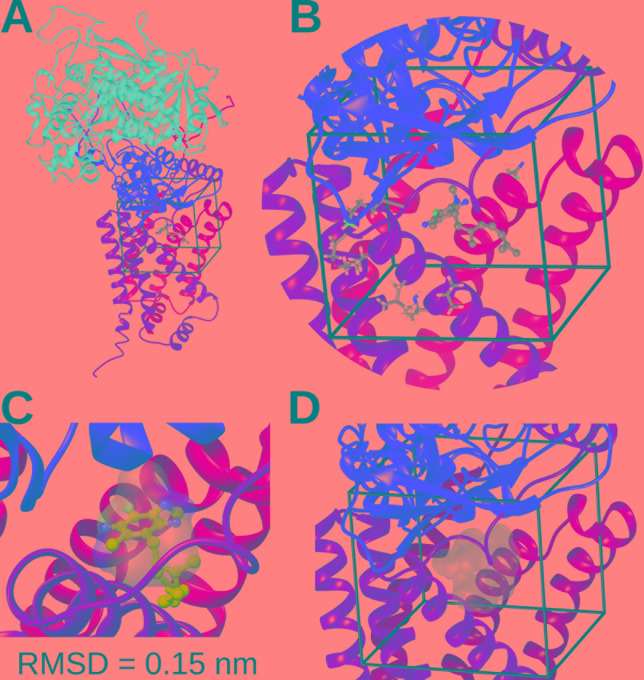
Complex II quinone-binding site. **(A)** Cartoon representation of the adult *A. suum* complex II crystallized structure bound to RQ_2_ (i.e., with two isoprenoid units). Color code: Purple = SDHA; red = SDHB; orange = SDHC; yellow = SDHD. **(B)** Inset showing the docking search space (black lines), RQ_2_ (spheres and sticks representation), and nonconserved residues among *A. suum* and *C. elegans* included in the search space (tubes). **(C)** Comparison between the best docking solution for RQ_2_ (cyan spheres and sticks) and its binding site obtained from X-ray crystal structure (transparent surface). The best docking solution showed a root mean square deviation (RMSD) of 0.15 nm. **(D)** Superimposition of the best docking solutions for UQ_2_ and RQ_2_ performed for *C. elegans* and both adult and larva *A. suum* complex II models (transparent surface).

We performed *in silico* docking analysis in order to explore how the change in subunits of complex II may affect the potential binding modes of different quinones. To this end, three structures were used: the crystal structure of *A. suum* complex II purified from adult worms (PDB: 5C2T) and homology models of complex II obtained for *A. suum* larval stages and *C. elegans*. The latter encodes one gene for each subunit involved in binding quinones (SDHB, SDHC, and SDHD). The search space, indicated by a box with black edges in **Figure 4A**, was defined considering the binding site of RQ_2_ (*i.e.* RQ with two isoprene units) in 5C2T and giving sufficient volume to explore alternative binding modes. Multiple sequence alignments were performed on the SDH subunits of *A. suum* (adult and larval stages) and *C. elegans*. Results indicated that, within the docking search space, only eight amino acids are not conserved (six for SDHC and two for SDHD, **Supplementary Figure 2**). Those residues face oppositely to the ligand and are not involved in direct interactions with RQ_2_ (**Figure 4B**).

Docking calculations employing the three complex II models showed that the binding modes with higher affinities for both UQ_2_ and RQ_2_ fit without any steric impediment into the quinone binding pocket (**Figure 4D**). These results suggest that SDHB, SDHC, and SDHD gene duplications would not reflect an adaptation to the binding site of UQ or RQ.

**Figure 5:**
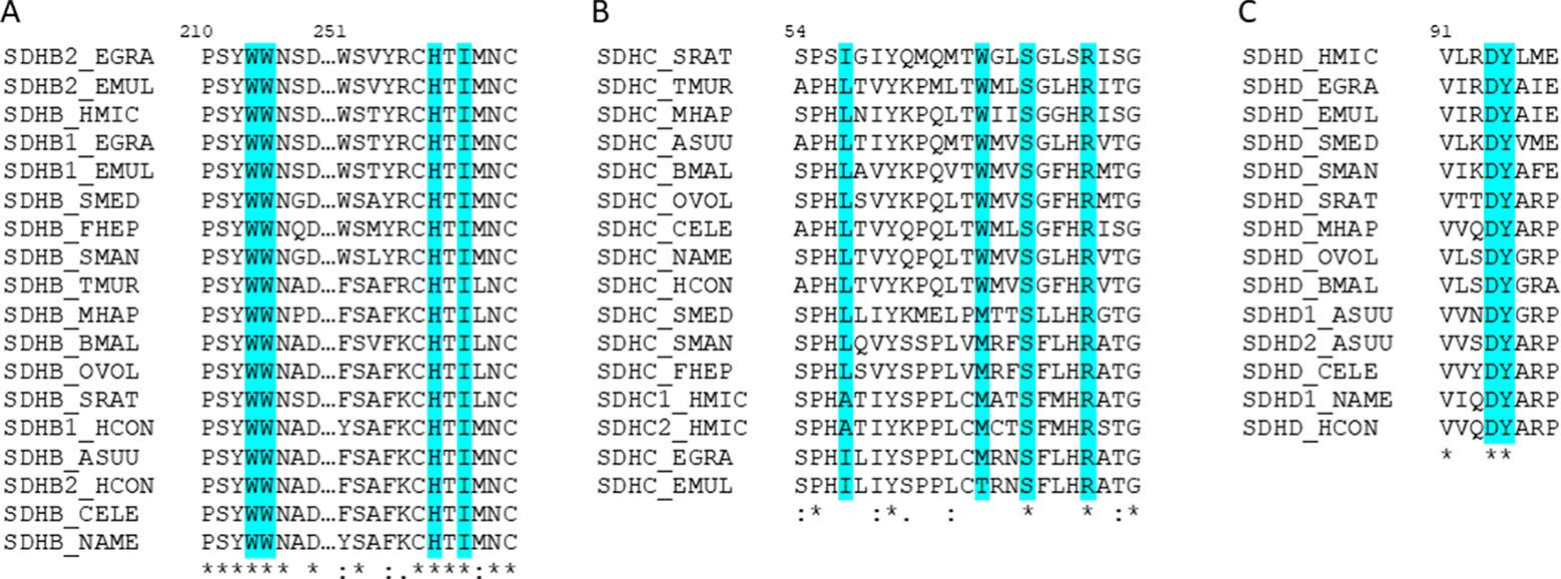
The amino acids interacting with quinones in complex II are highly conserved. Alignment of helminths’ complex II sequences containing the identified amino acids involved in quinone-binding in **(A)** SDHB, **(B)** SDHC, and **(C)** SDHD. In turquoise are highlighted the residues identified as interacting with quinones. The numeration corresponds to *C. elegans* sequences.

We then analyzed the residues involved in complex II quinone binding in nematodes and platyhelminths. We found that the residues of SDHB and SDHD are conserved in all the analyzed species. SDHC allows some changes in quinone-binding positions (**Figure 5**). Interestingly, in both *H. microstoma* SDHC, the only species that has two *sdhc*, the quinone-binding positions are conserved.

### *sdha-1* and *sdha-2* as Well as *nduf2-1 and nduf-2.2* Showed an Inverted Expression Profile During *C. elegans* Lifecycle

**Figure 6:**
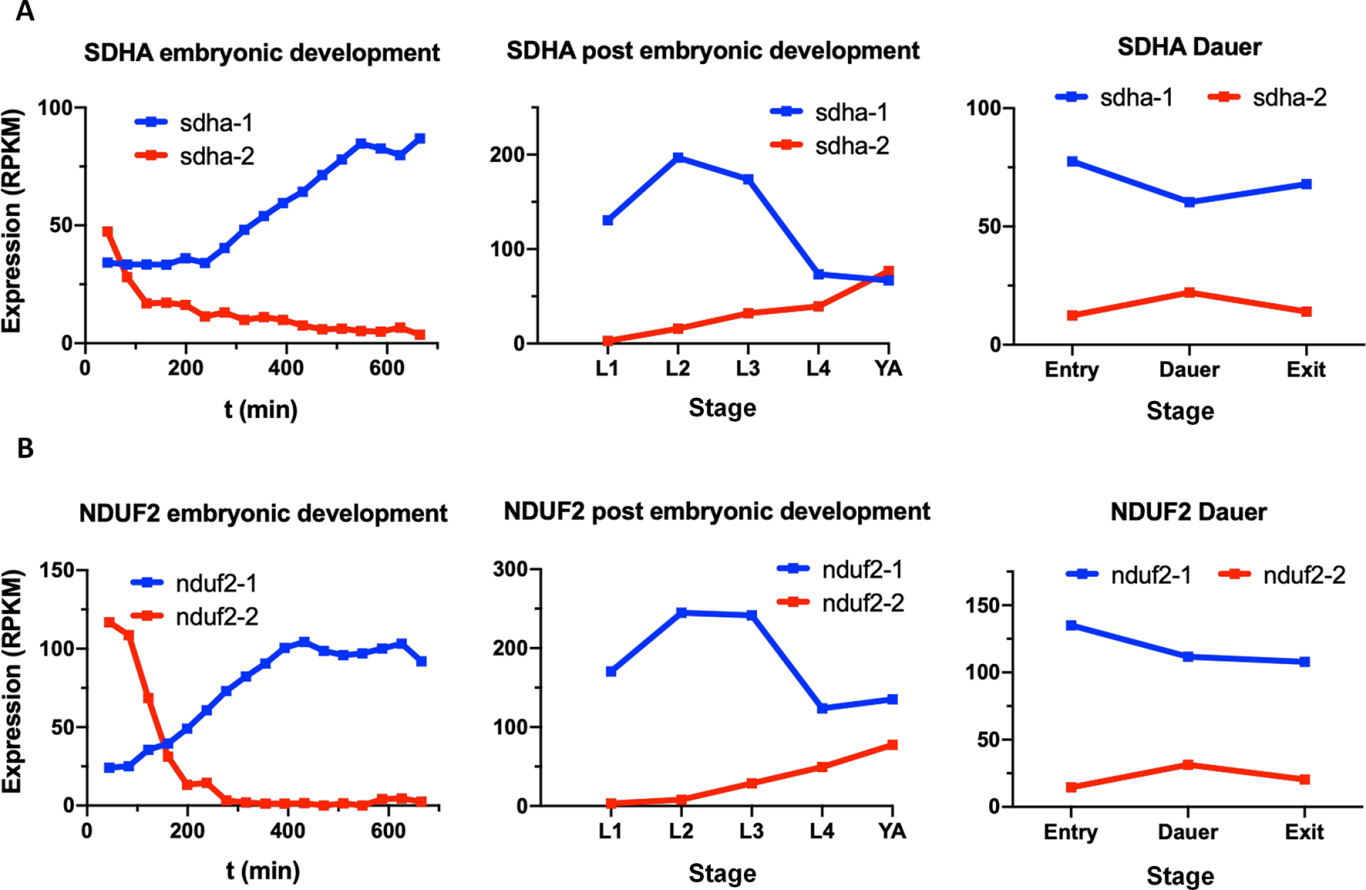
Expression of *sdha* and *nduf2* genes throughout *C. elegans* lifecycle. Expression of *sdha-1* and *sdha-2* **(A)**, and *nduf2-1* and *nduf2-2* **(B)** during *C. elegans* development. Expression during embryonic development is shown on the left graphs. Egg laying occurs around 150 min after fertilization. The four larval stages (L1, L2, L3, and L4) and young adults are shown in the middle graphs. The expression levels for dauer larvae at three timepoints (entry, dauer, and exit) are shown on the right graphs.

In the *C. elegans* genome, there are two *sdha* and two *nduf2* genes, as in *A. suum*. *C. elegans* SDHA-1 is essential and has the highest level of homology to *A. suum* SDHA-1 (larval SDHA) and *C. elegans* SDHA-2 to *A. suum* SDHA-2 (adult SDHA). In the case of NDUF2, NDUF2-1 is essential, and both *C. elegans* proteins (NDUF2-1 and NDUF2-2) are more similar to each other than to any NDUF2 proteins of *A. suum*. We analyzed the expression of *sdha* and *nduf2* during *C. elegans* lifecycle. The results are shown in **Figure 6**. In general, *sdha-1* has a higher expression than *sdha-2*, and both genes show an inverted expression profile along the different developmental stages. *sdha-1* expression increases towards the end of the embryonic development and is highest during larval stages L2–L3 and then decreases in the last larval stage (L4) and in the adult worm (**Figure 6A**). In contrast, *sdha-2* expression is highest during the early embryo and then starts to decrease near L1 larval stage. Its expression increases again towards the adult worm (**Figure 6A**). *C. elegans* has an alternative larval stage named dauer, which allows it to endure when environmental conditions are not adequate for normal growth (Cassada and Russell, 1975). After entry in dauer, *sdha-1* expression decreases while *sdha-2* expression increases (**Figure 6A**).

Similar to *sdha-1* and *sdha-2*, *nduf2-1* expression is higher than *nduf2-2* along *C. elegans* lifecycle, except at the beginning of the embryo development. The expression patterns of *nduf2-1* and *nduf2-2* echo that of *sdha-1* and *sdha-2*, respectively, in the embryo, larval and adult stages, as well as in the dauer larvae (**Figure 6B**). Interestingly, during embryonic development, *nduf2-1* and *nduf2-2* expression curves cross each other around the time of egg laying.

These observations suggest that there is a tight regulation of the expression of SDHA and NDUF2 coding genes throughout the lifecycle of *C. elegans*.

### Progeny Is Reduced in *sdha-2 and nduf2-2* Mutant Worms

**Figure 7:**
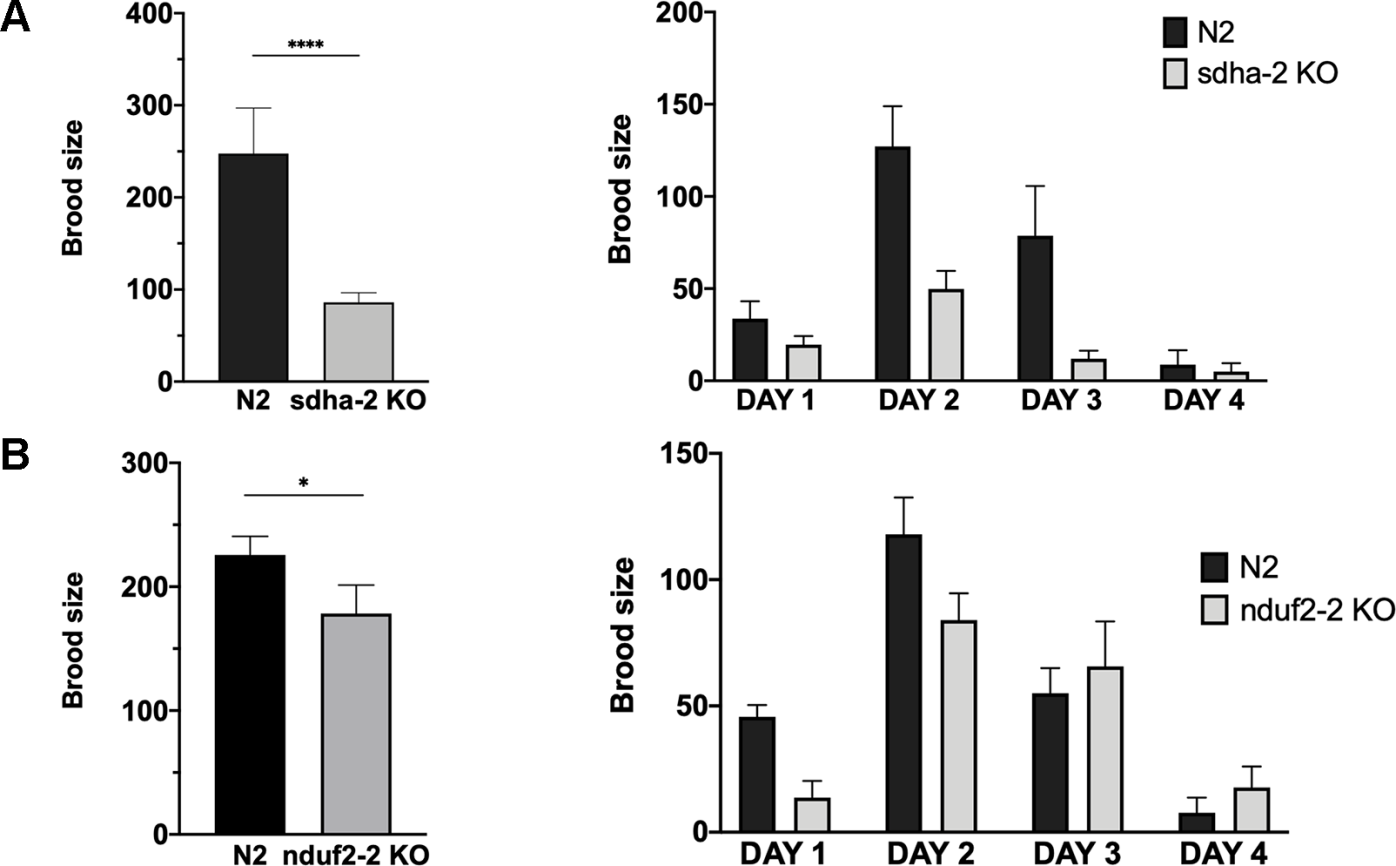
Brood size of *sdha-2* and *nduf2-2* KO worms is decreased in normoxia. Total progeny was analyzed for 45 worms for both strains, derived from three independent experiments with 15 worms each. The total progeny per worm is represented on the left and progeny/worm/day on the right. **(A)** *sdha-2* KO strain, **(B)** *nduf2-2* KO strain. Asterisks represent a statistically significant difference between two strains. *p < 0.05; ****p < 0.0001.

As already mentioned, the expression of *nduf2-2* and *sdha-2* increases in the adult worm and is highest in the early embryo. Thus, we assessed the effect of the absence of these genes on the reproductive potential of the worm. The analysis of the brood size of KO strains in *nduf2-2* and *sdha-2* showed that the total amount of offspring is significantly reduced in both KO worms compared to the wild-type strain (N2) (**Figure 7**); this reduction was more pronounced in the *sdha-2* KO worms. We also observed a few hours delay in development from L1 to the beginning of egg laying in *nduf2-2* KO worms compared to N2.

Since in *A. suum* a duplication in the SDHA coding gene has been associated with an adaptation to environments with different oxygen tensions, we examined whether the duplications in *sdha* and *nduf2* in *C. elegans* could also be related to changes in oxygen availability. Similar to N2, *nduf2-2* and *sdha-2* KO strains survived after 24 h exposure to hypoxia (0.4% O_2_) or anoxia. We then tested brood size for these strains after a 24-h exposure to hypoxia or anoxia. The phenotype previously observed under normoxic conditions was not exacerbated under hypoxia or anoxia for these mutants (**Supplementary Figure 4**).

Our results indicate that *sdha-2* and *nduf2-2* are not essential for fecundity, but the absence of these genes reduces progeny production of the worm.

## Discussion

RQ has been identified in all nematodes and platyhelminths that have been analyzed for its presence. Owing to its redox potential (intermediate between complex I and II), RQ is thought to be the key metabolite in the alternative ETC used under hypoxic conditions. Until now, it was thought that the use of RQ in helminths required alternative complex II subunits derived from duplicated genes. Our results contradict this view. Indeed, some species do not have any complex II gene duplications (e.g., *Meloidogyne hapla*, *Strongyloides ratti*, *Necator americanus*, *S. mediterranea*, and *Schistosoma mansoni*), while others have even lost *sdhd* (*F. hepatica* and *T. muris*). Furthermore, there is not a unique evolutionary event associated with the use of RQ in complex I and II genes. We examined several life history traits for the different species whose genomes were analyzed. In particular, we examined absence/presence of free-living stage(s), parasitized host(s) (intermediate, definitive, vector), parasitized site/tissue, and reproduction mode (monoecious vs. dioecious). We have not found any particular association of life history traits or style that could explain a trend regarding the presence of alternative ETCs in the different lineages. Thus, RQ has not been a driving force in complex II gene history. Although RQ is a metabolic signature for hypoxic metabolism, this metabolite is not necessarily associated with a change in complex I and II composition. Nevertheless, the variety of complex II genes duplications observed in nematodes and platyhelminths suggests that different adaptations have occurred in different lineages.

Previous studies in *A. suum* have shown that both duplicated complex II subunits (SDHA and SDHD) are exchanged when the environment and oxygen tension change. These adjustments are responsible for the change in the SDH-FRD enzymatic activity and have been associated with the need to use different quinones (Tielens and Van Hellemond, 1998; Amino et al., 2000; Hellemond et al., 2003; Iwata et al., 2008; Harada et al., 2013; ). In our study, we show that both quinones could bind to both *A. suum* SDHD subunits. Moreover, in organisms that have duplicated the subunits that contact quinones (SDHB, SDHC, and SDHD), no changes are observed in the amino acids that interact directly with quinones. Thus, the switch of subunits does not appear to be related to the quinone-binding capacity.

Gene duplications in complex I have not been previously examined in nematodes and platyhelminths. We searched for the nuclear-encoded quinone-binding subunit genes (*nduf2* and *nduf7*) and found that different gene duplication events occurred among different lineages, similar to the phenomenon observed for complex II subunits. So far, these gene duplications have not been associated with any particular phenotype or adaptation. Residues in NDUF2 and NDUF7 that interact with quinones have been identified in a previous study (Degli Esposti, 2015). This study identified a Tyr residue in *A. suum* NDUF2-1 that differs from Lys or Arg residues found in all other NDUF2 analyzed. We propose that this would be an adaptation of nematodes to RQ binding. Although we found that Tyr is the most frequent amino acid, His and Lys can also be found at this position (**Supplementary Figure 3**).

The preferred oxygen concentration for the free-living nematode *C. elegans* is around 7–14% (Chang and Bargmann, 2008). *C. elegans* possesses complex I and II subunit duplications. To address the role of duplicated complex I and II subunits in this organism, we analyzed their expression pattern during its lifecycle and observed developmental regulation of their expression. A striking observation is that the essential subunits NDUF2-1 and SDHA-1 (those for which KO is lethal) show a similar expression pattern, which is inverted to the expression pattern of the nonessential subunits (NDUF2-2 and SDHA-2). The essential subunits are generally more highly expressed than the nonessential ones, except in the adult worm and early embryo, suggesting that the nonessential subunits are relevant during these stages. Consistent with this interpretation, the *nduf2-2* and *sdha-2* KO strain have reduced brood size compared to the wild-type strain N2. It is worth noting that *nduf2* and *sdha* duplications co-occur in nematodes. Our results suggest that the duplicated subunits could be part of alternative ETC complexes needed to meet different metabolic demands during the lifecycle of the worm and serves to increase the overall organismal fitness. In this sense, it is worth noting that expression of *nduf2-2* and *sdha-2* increase in the early embryo and dauer stage, where a hypoxic metabolism is thought to occur.

The association of an alternative complex II with RQ has been firmly established in *A. suum*, becoming a paradigm in helminth biochemistry. However, our results highlight that *A. suum* is the exception rather than the rule. Furthermore, different variations in the complex I subunits that interact with quinones are also observed in distinct helminth lineages. Dissimilar subunit arrays for ETC complexes appear to have evolved to adapt to different stages of helminth lifecycles, environmental conditions, or to specific cells or tissues within worms.

## Material and Methods

### Sequences Identification and Analysis

In order to obtain information about helminth complex II and nuclear-encoded quinone-binding complex I subunits, we analyzed genomes and transcriptomes of six platyhelminths (*E. granulosus*, *E. multilocularis*, *H. miscrostoma*, *F. hepatica*, *S. mansoni*, and *S. mediterranea*) and nine nematodes (*T. muris*, *A. suum*, *Brugia malayi*, *Onchocerca volvulus*, *M. hapla*, *S. ratti*, *H. contortus*, *N. americanus*, and *C. elegans*). Sequences were retrieved from https://parasite.wormbase.org (WBPS10), UniProt (https://www.uniprot.org) and *S. mediterranea* database (http://smedgd.neuro.utah.edu).

Searches were performed initially with Protein Basic Local Alignment Search Tool (BLASTP) (protein databases) using *A. suum* and *C. elegans* SDHA, SDHB, SDHC, SDHD, NDUF2, and NDUF7 sequences as queries and confirmed by best reciprocal hits in BLAST. Additionally, TBLASTN searches were performed using genomic sequences and cDNAs databases and the protein sequences previously identified or the most closely related organism’s sequences as queries. This served to confirm the annotated protein sequences and to identify the nonannotated ones. Identified sequences were confirmed by best reciprocal hits in BLAST.

To study the evolutionary history of complex II and nuclear-encoded quinone-binding complex I subunits, phylogenetic analysis were performed in the website phylogeny.fr (Dereeper et al., 2008; Dereeper et al., 2010) for every set of identified subunits. Multiple sequence alignments were made with MUSCLE 3.8 (Chojnacki et al., 2017) and then curated with Gblocks 0.91b (Castresana, 2000;Talavera and Castresana, 2007). Phylogeny analyses were made with PhyML 3.0 (Guindon et al., 2010) and tree rending with TreeDyn 198.3 (Chevenet et al., 2006; Dereeper et al., 2008). Alignments for all subunits studied are available in **Supplementary Figure 1**.

### Modeling of Complex II Structures

Complex II structures for *A. suum* (normoxia) and *C. elegans* were built by homology modeling with Modeller (Webb and Sali, 2016), using as a 3D template the X-ray structure of *A. suum* (hypoxia) stored in the Protein Data Bank with the code 5C2T. Multiple sequence alignment of complex II between *A. suum* (normoxia and hypoxia) and *C. elegans* was done with Clustal Omega (Chojnacki et al., 2017). To validate the docking technique, we compared the predicted binding sites for RQ_2_ with the one from the crystallized structure 5C2T. The best docking solution showed a root mean square deviation of 0.15 nm (**Figure 4C**). A 0.2-nm root mean square deviation cutoff is often used as a criterion of the correct bound structure prediction (Bursulaya et al., 2003).

Docking calculations were performed with AutoDock Vina 1.1.2 (Trott and Olson, 2010). Input PDBQT files for the receptors and ligands were prepared with AutoDockTools 1.5.6 (Morris et al., 2009). All torsions of the ligand were set as fully rotatable, applying to the receptors a partially flexible treatment. The maximum number (10) of rotatable side chains allowed by the program was chosen, selecting residues located closer than 0.6 nm from RQ_2_ in 5C2T. List of flexible residues by domain: SDHB: Trp196, Trp197, His240, Ile242; SDHC: Leu60, Trp69, Ser72, Arg76; SDHC: Asp106 and Tyr 107. The search space was defined by a grid with *x*, *y*, and *z* dimensions of 26 × 24 × 24 Å. The grid was centered so that the binding pocket corresponding to RQ_2_ could be included in the search space.

### Expression Profile Analysis of SDHA and NDUF2 Coding Genes

Transcriptomic data used for this analysis were taken from the GExplore web (Hutter and Suh, 2016).The values of depth of coverage per million bases for each time-point were converted to reads per kilobase per million mapped reads to use a more familiar representation of expression values.

### Strains and Culture Conditions

*C. elegans* wild-type Bristol N2 (N2) and VC393 (*nduf2-2* (*ok437*)III) were provided by the *Caenorhabditis* Genetics Center. VC393 has a 1,448-bp deletion expanding several exons of *nduf2-2*. *C. elegans* TM1420 (*sdha-2* (*tm1420*) I) was obtained from the National BioResource Project. This strain has a 519-bp deletion in exon 4 of *sdha-2*.

Worms were maintained at 20°C on nematode growth medium agar plates seeded with *Escherichia coli* OP50. To obtain synchronized worms, gravid adults on nematode growth medium agar plates were treated with an alkaline hypochlorite solution, and eggs were collected and placed in M9 medium without food, at 20°C for 24 h (Brenner, 1974).

### Brood Size Analysis in *sdha-2 and nduf2-2* KO Strains

Wild-type (N2) and mutant (*sdha-2* KO and *nduf2-2* KO) worms were synchronized, and, after reaching the L4 stage, 15 worms for each strain were separated and plated in groups of 3. When a phenotype was observed, the experiment was performed three times. Offspring production was counted every day for 4 days.

## Data Availability Statement

All datasets generated for this study are included in the article/**Supplementary Material**.

## Author Contributions

LO performed the genomic and phylogenetic analyses. CM-R performed the expression analysis and the experiments with *C. elegans* strains. EB and SP performed the molecular modeling studies of complex II with quinones. LO, CM-R, EB, SP, and GS analyzed the data, drafted, and wrote the manuscript. GS conceptualized the study.

## Funding

This study was supported by Agencia Nacional para la Innovación y la Investigación (ANII, FCE_1_2017_1_136340) and Ministerio de Educación y Cultura (MEC, FVF 2017/030). This work was partially funded by FOCEM (MERCOSUR Structural Convergence Fund), COF 03/11. CM was recipient of a fellowship from ANII (FCE_1_2014_1_104366). EB is beneficiary of a postdoctoral fellowship of Consejo Nacional de Investigaciones Científicas y Técnicas, Argentina (CONICET).

## Conflict of Interest

The authors declare that the research was conducted in the absence of any commercial or financial relationships that could be construed as a potential conflict of interest.

## Abbreviations

FRD, fumarate reductase; RQ, rhodoquinone; SDH, succinate dehydrogenase; UQ, ubiquinone.
