# Supplementary material for: Complex I and II Subunit Gene Duplications Provide Increased Fitness to Worms
Source: Front Genet. 2019 Oct 25;10:1043. doi: 10.3389/fgene.2019.01043 (PMC6859908; doi:10.3389/fgene.2019.01043)
Supplement: Supplementary file 1 [file Image_1.pdf]

SDHA

```
SDHA_TMUR       -------------------------------MTSSIQHAQLPRHV---------------
SDHA_SMED       MSRLLFNLKNICGIGLNKCSIGKFSTSGKLSATQISKDYTVIDHVYDAVVVGAGGAGLRA
SDHA_EGRA       -------------------------------VSTVGKDYTIIDHTYDAVVVGAGGAGLRA
SDHA_EMUL       -------------------------------VSTVGKDYTIIDHTYDAVVVGAGGAGLRA
SDHA_HMIC       -------------------------------VSTVGKDYTIIDHTYDAVVVGAGGAGLRA
SDHA_FHEP       -------------------------------AGSYSKDYTVIDHTFDAVVVGAGGAGLRA
SDHA_SMAN       ----------------------------------------IIDHSFDAVVVGAGGAGLRA
SDHA2_BMAL      -----------------------------RAKKTYIANYTIIDHNYDAIVVGAGGAGLRA
SDHA2_OVOL      -------------------------------KTTNISNYTVVDHNYDAVVVGAGGAGLRA
SDHA1_ASUU      -----------------------------DVSTSNIAQYKVIDHAYDVVIIGAGGAGLRA
SDHA2_CELE      ---------------------KTPVRTYMKKQVSATTNFDVVDHTFDAVVVGAGGAGLRA
SDHA_NAME       ----------------------ALAASTTDAKHGNIANYKVVDHAYDAVVVGAGGAGLRA
SDHA_HCON       -----------------------------DAKRGNIANYKVVDHAFDAVVVGAGGAGLRA
SDHA1_BMAL      -----------------------------GTRTSNIAEYCVVDHAYDAVVVGAGGAGLRA
SDHA1_OVOL      -----------------------------EAKTSSIGEYHIVDHAFDSVVVGAGGAGLRA
SDHA_SRAT       -----------------------------CAKKGNISDYKVIDHTFDAVVVGAGGAGLRA
SDHA_MHAP       ------------------------------------------DHTFDAVVVGAGGAGLRA
SDHA1_CELE      --------------------VSLSAANHSDAKRSDIAQYKVVDHAYDAVVVGAGGAGLRA
SDHA2_ASUU      -----------------------------AAKTSNIAEYKVVDHAFDAVVVGAGGAGLRA
                                                           *

SDHA_TMUR       ------ERKRC-------------------------------SKAATTQGGINAALGNME
SDHA_SMED       AFGLANSGFKT---------------------ACITKLFPTRSHTVAAQGGINAALGNME
SDHA_EGRA       AFGLANEGFKT---------------------ACVTKLFPTRSHTVAAQGGINAALGNME
SDHA_EMUL       AFGLANEGFKT---------------------ACVTKLFPTRSHTVAAQGGINAALGNME
SDHA_HMIC       AFGLSNEGFKT---------------------ACVTKLFPTRSHTVAAQGGINAALGNME
SDHA_FHEP       AFGLANEGFKT---------------------ACITKLFPTRSHTVAAQGGINAALGNME
SDHA_SMAN       GFGLANEGFKT---------------------AIITKLFPTRSHTVAAQGGINAALGNME
SDHA2_BMAL      AARLGEGGLRV---------------------AVITKLFPTRSHTVAAQGGINAAIGSMN
SDHA2_OVOL      ASRLGEGGLRV---------------------AIITKLFPTRSHTVAAQGGINAAIGSMN
SDHA1_ASUU      AMGLGEAGFKT---------------------AVVTKMFPTRSHTTAAQGGINAALGSMN
SDHA2_CELE      AMGLSEGGMKT---------------------AVITKLFPTRSHTVAAQGGVNAALGNMN
SDHA_NAME       AMGLSEGGLKT---------------------AVITKIFPTRSHTVAAQGGINAAIGSMN
SDHA_HCON       AMGLSEGGLKT---------------------AVITKIFPTRSHTVAAQGGINAALGSMN
SDHA1_BMAL      AMGLSEGGQKV---------------------AVVTKLFPTRSHTVAAQGGVNAALGNMN
SDHA1_OVOL      AMGLSEGGQNV---------------------AVITKLFPTRSHTVAAQGGVNAALGNMN
SDHA_SRAT       AMGLAEGGMKT---------------------AVITKLFPTRSHTVAAQGGINAALGNMN
SDHA_MHAP       AMGLAEGGMKTGVCILIKKDSVNKHLILSCILAVVTKLFPTRSHTVAAQGGINAALGNMN
SDHA1_CELE      AMGLAEGGLKT---------------------AVITKLFPTRSHTVAAQGGINAALGNMN
SDHA2_ASUU      AMGLSEGGLKT---------------------AVITKLFPTRSHTVAAQGGVNAALGNMN
                         .                                *::.::***:***:*.*:

SDHA_TMUR       PDNWRWHFYDTVKGSDWLGDQDAIHYMCREAPRAVLELENYGMPFSRTKEGRIYQRAFGG
SDHA_SMED       KDDWRFHMYDTVKGSDWLGDQDAIHYMCEEAPKTVIELENYGMPFSRLDNGMIYQRAFGG
SDHA_EGRA       QDHWKFHMFDTVKGSDWLGDQDAIHYMCEEAPKAVIELENYGMPFSRLENGKIYQRAFGG
SDHA_EMUL       QDHWKFHMFDTVKGSDWLGDQDAIHYMCEEAPKAVIELENYGMPFSRLENGKIYQRAFGG
SDHA_HMIC       KDDWKFHMFDTVKGSDWLGDQDAIHYMCEEAPKAVIELENYGMPFSRLENGKIYQRAFGG
SDHA_FHEP       NDDWRYHMYDTVKGSDWLGDQDAIHYMCEEAPKAVIELENYGMPFSRLENGKIYQRAFGG
SDHA_SMAN       PDDWRFHMYDTVKGSDWLGDQDAIHYMCEEAPKAVIELENYGVPFSRLENGKIYQRAFGG
SDHA2_BMAL      PDNWKWHFYDTVKGSDWLGDQNAIHYLTKDAVRAVIELENYGMPFSRTAEGKIYQRSFGG
SDHA2_OVOL      PDDWKWHFYDTVKGSDWLGDQNAIHYLTKDAVRAVIELENYGMPFSRTAEGKIYQRSFGG
SDHA1_ASUU      PDDWKWHFYDTVKGSDWLGDQNAMHYLTRNAVEAVTELENFGMPFSRTPEGKIYQRSFGG
SDHA2_CELE      PDNWRWHFYDTVKGSDWLGDQDAIHYMTREAERAIIELENYGMPFSRTTDGKIYQRAFGG
SDHA_NAME       KDDWRWHFYDTVKGSDWLGDQDAIHYMCREACRAVIELENYGMPFSRTADGKIYQRAFGG
SDHA_HCON       KDDWRWHFYDTVKGSDWLGDQDAIHYMTREACRAVIELENYGMPFSRTPEGKIYQRAFGG
SDHA1_BMAL      PDDWRWHFYDTVKGSDWLGDQNAIHYMTREAVRAVIEMENYGMPFSRTEEGKIYQRSFGG
```

```
SDHA1_OVOL      PDDWRWHFYDTVKGSDWLGDQNAIHYMTREAVRAVIEMENYGMPFSRTEEGKIYQRSFGG
SDHA_SRAT       KDDWRWHFYDTVKGSDWLGDQDAIHYMTREAPRAVIELENYGMPFSRTEDGKIYQRAFGG
SDHA_MHAP       PDDWRWHFYDTVKGSDWLGDQDAIHYMTREAPRAVIELENYGMPFSRTEGGKIYQRAFGG
SDHA1_CELE      PDNWRWHFYDTVKGSDWLGDQDAIHYMTREAERAVIELENYGMPFSRTTDGKIYQRAFGG
SDHA2_ASUU      PDDWRWHFYDTVKGSDWLGDQDAIHYMTREAVRAVIELENYGMPFSRTADGKIYQRAFGG
                 * *.:*::************:*:**:  :*  :: *:**:*:****   * ****:***

SDHA_TMUR       QSLNFGTGGQAHR---TCCVADRTGHSMLHTLYGRTLAYDCTYFLEYFAMDLLMDKGRCN
SDHA_SMED       QAIDYGKGGQAHR---CCCVADRTGHSLLHTLYGRSLKYDTDYFIEYFAMDLIMENGECR
SDHA_EGRA       QSLDYGKGGQAHR---CCAVADRTGHSLLHTLYGRSLRYETDYFIEYFALDLLMENGACR
SDHA_EMUL       QSLHYGKGGQAHR---CCAVADRTGHSLLHTLYGRSLRYETDYFIEYFALDLLMENGVCR
SDHA_HMIC       QSLDYGKGGQAHR---CCAVADRTGHSLLHTLYGRSLRYETDYFVEYFALDLLMENGVCR
SDHA_FHEP       QSIDYGRGGQAHR---CCAVADRTGHSLLHTLYGRSLRYDTTYFVEYFAMDLLMENGACR
SDHA_SMAN       QSIDYGRGGQAHR---CCAVADRTGHSLLHTLYGRSLRYDATYFIEYFVLDLLMENGECR
SDHA2_BMAL      QSNNYGKGGVAKR---TCAVADRTGHSMLHTLYGTSLQFHCDYYIEFFALDLLMSDKRCI
SDHA2_OVOL      QSNEYGRGGVAKR---TCAVADRTGHAMLHTLYGTSLQFHCHYYIEFFALDLLMDKERCV
SDHA1_ASUU      QSNNYGKGGVAKR---TCCVADRTGHSMLHTLYGNSLRCHCTFFIEYFALDLLMDKGRCV
SDHA2_CELE      QSNDFGRGGQAHR---TCCVADRTGHSLLHTLYGASLQYDCNYFVEYFALDLIMDKGKCI
SDHA_NAME       QSNDFGRGGQAHR---TCAVADRTGHSLLHTLYGASLQYNCNYFVEYFALDLIMDKGVCV
SDHA_HCON       QSNDYGRGGQAHR---TCAVADRTGHSLLHTLYGASLQYNCNYFVEYFALDLIMDKGACV
SDHA1_BMAL      QSNNFGKGGMARR---TCCVADRTGHSMLHTLYGSSLQYNCRYFIEYFALDLLMDNGRCV
SDHA1_OVOL      QSNNFGKGGMARR---TCCVADRTGHSMLHTLYGSSLQYNCRYFIEYFALDLLMDKGRCV
SDHA_SRAT       QSNDFGRGGQAHR---TCCVADRTGHSLLHTLYGASLQYDCKYFIEYFALDLIMDKGECV
SDHA_MHAP       QSNDFGRGGQAHR---TCCVADRTGHSLLHTLYGASLQYSCQYFVEYFALDLIMDQERCV
SDHA1_CELE      QSNDFGRGGQAHR---TCCVADRTGHSLLHTLYGASLQYNCNYFVEYFALDLIMENGVCV
SDHA2_ASUU      QSNDFGRGGQAHRQNMTCCVADRTGHSMLHTLYGSSLQYNCQYFIEFFALDLIMDKGACV
                *:  :* ** *.*    *.*******::****** :*     :::*:*.:**:*..  *

SDHA_TMUR       GVIALNLEDGSIHRFRTANTILATGGYGRAYFSCTSAHTCTGDGTAMVTRAGLPNSDMEF
SDHA_SMED       GVIALNLEDGTIHRFKSKNTVLATGGYGRSFFSCTSAHTCTGDGTAMVSRAGLPNQDMEF
SDHA_EGRA       GINAMCLEDGTIHRFRAKNTILATGGYGRAYFSCTSAHTCTGDGTAMVTRAGLPNEDMEF
SDHA_EMUL       GINAMCLEDGTIHRFRAKNTILATGGYGRAYFSCTSAHTCTGDGTAMVTRAGLPNEDMEF
SDHA_HMIC       GVNAICLEDGTIHRFRAKNTILATGGYGRAYFSCTSAHTCTGDGTAMVSRAGLPNEDMEF
SDHA_FHEP       GVIAMCLEDGSIHRFRAKNTVLATGGYGRTYFSCTSAHTCTGDGTAMITRAGLPNEDMEF
SDHA_SMAN       GVIAVCLEDGTLHRFRSKNTVLATGGYGRTYFSCTSAHTCTGDGTAMVTRAGLPNQDMEF
SDHA2_BMAL      GIIAMNLEDGTFHRFRAPFTVLATGGFGRAYFSCTTAHSTTGDGNAMVIRAGLQTTDMEF
SDHA2_OVOL      GVIAMNLEDGTFHRFQAPFTVLATGGFGRAYFNCTTAHSTTGDGNAMAIRAGLQTTDMEF
SDHA1_ASUU      GVIALCLEDGTIHRFRSKRTIVATGGYGRAYFSCTTAHMNTGDGTALATRAGIALEDLEF
SDHA2_CELE      GVVALDIETGQIHRFRAKNTVLATGGYGRAYFSCTSAHTCTGDGTALTARAGIRNSDMEF
SDHA_NAME       GVVALCMEDGTIHRFRSKNTVLATGGFGRAYFSCTSAHTCTGDGTALVARAGISNTDMEF
SDHA_HCON       GVVAMCLEDGTIHRFRSKNTVLATGGYGRAYFSCTSAHTCTGDGTGMVARAGINNTDMEF
SDHA1_BMAL      GIIAMDLEDGSIHRFRAKNTVVATGGYGRAFFSCTSAHTCTGDGTAMITRAGLQNSDMEF
SDHA1_OVOL      GIIAMNLEDGSIHRFRAKNTVIATGGYGRAFFSCTSAHTCTGDGTAMVARAGLQNSDMEF
SDHA_SRAT       GVIAMDLEDGSIHRFNAKNTVLATGGYGRAYFSCTSAHTCTGDGTAMVARAGLANSDMEF
SDHA_MHAP       ------------------------------------------------------------
SDHA1_CELE      GVIAMDLEDGTIHRFRSKNTVLATGGYGRAFFSCTSAHTCTGDGTALTARAGINNSDMEF
SDHA2_ASUU      GVVAMDLEDGTIHRFRSKNTVLATGGYGRAFFSCTSAHTCTGDGTALATRAGIGNSDMEF

SDHA_TMUR       VQFHPTGIYGAGCLITEGSRGEGGFLVNSKGERFMERYAPNAKDLASRDVVSRAMAIEIR
SDHA_SMED       IQFHPTGIYGAGCLITEGCRGEGGYLINSEGERFMERYAPKAKDLASRDVVSRSMTIEIR
SDHA_EGRA       VQFHPTGIYGAGCLITEGCRGEGGYLINSEGERFMERYAPNAKDLASRDVVSRAMTIEIR
SDHA_EMUL       VQFHPTGIYGAGCLITEGCRGEGGYLINSEGERFMERYAPNAKDLASRDVVSRAMTIEIR
SDHA_HMIC       VQFHPTGIYGAGCLITEGCRGEGGYLINSEGERFMERYAPNAKDLASRDVVSRAMTIEIR
SDHA_FHEP       VQFHPTGIYGAGCLITEGCRGEGGYLVNSQGERFMERYAPNAKDLASRDVVSRSMTIEIR
SDHA_SMAN       VQFHPTGIYGAGCLITEGCRGEGGYLINSKGERFMERYAPNAKDLASRDVVSRAMTIEIR
SDHA2_BMAL      IQFHPTGIYGVGCLITEGSRGEGGYLVNSKGERFMKKYAPKALDLASRDVVSRAMTIEII
```

```
SDHA2_OVOL      IQFHPTGIYGVGCLITEGSRGEGGYLVNSKGERFMKKYAPKALDLASRDVVSRAMTIEII
SDHA1_ASUU      IQFHPTGIYGVGCLITEGSRGEGGFLVNSEGERFMERYAPKAKDLASRDVVSRAETIEIM
SDHA2_CELE      VQFHPTGIYGVGCLITEGSRGEGGYLVNSQGERFMERYAPNAKDLASRDVVSRAMTMEIN
SDHA_NAME       VQFHPTGIYGAGCLITEGSRGEGGYLVNSKGERFMERYAPNAKDLASRDVVSRAMTVEVM
SDHA_HCON       VQFHPTGIYGAGCLITEGSRGEGGFLVNSKGERFMERYAPNAKDLASRDVVSRAMTVEVM
SDHA1_BMAL      VQFHPTGIYGAGCLITEGSRGEGGYLVNSEGERFMKKYAPNALDLASRDVVSRAMTIEIM
SDHA1_OVOL      VQFHPTGIYGAGCLITEGSRGEGGFLVNSEGERFMKKYAPNALDLASRDVVSRAMTIEIM
SDHA_SRAT       VQFHPTGIYGAGCLMTEGCRGEGGFLVNSEGERFMERYAPVAKDLASRDVVSRSMTIEIM
SDHA_MHAP       --------------------GEGGFLINSEGERFMERYAPIAKDLASRDVVSRSMTLEIM
SDHA1_CELE      VQFHPTGIYGAGCLITEGSRGEGGYLVNSAGERFMERYAPNAKDLASRDVVSRSMTVEIM
SDHA2_ASUU      VQFHPTGIYGAGCLITEGSRGEGGFLVNSKGERFMERYAPNAKDLASRDVVSRAMTVEIM
                                    ****:*:** *****:.*** * **********: ::*:

SDHA_TMUR       EGRGVGPLKDHIYLQLHHLPAQLIHERLPGIAETAHVFAGVDCTKQPIPVLPTVHYNMGG
SDHA_SMED       EGRGVGPHKDHIYLQLSHLPADLLKQRLPGISETAMIFAGVDVTREPIPVLPTVHYNMGG
SDHA_EGRA       EGRGVGPRKDHCYLKLHHLPPDLLKSRLPGISETAEIFAGVDVTKEPVPVLPTVHYNMGG
SDHA_EMUL       EGRGVGPRKDHCYLKLHHLPPDLLKSRLPGISETAEIFAGVDVTKEPVPVLPTVHYNMGG
SDHA_HMIC       EGRGVGPRKDHCYLKLHHLPAEILKSRLPGISETAEIFAGVDVTKDPVPVLPTVHYNMGG
SDHA_FHEP       EGRGVGPRKDHIYLQLSHLPADQLHARLPGISETAKIFAGVDVTREPIPVLPTVHYNMGG
SDHA_SMAN       EGRGVGPRKDHIFLQLSHLPADQLHSRLPGISETAKIFAGVDVTRDPIPVLPTVHYNMGG
SDHA2_BMAL      EGRGVGKENDHIYLQLHHIPADYLHNKLPGIMETAKIFAGVDATKEPIPVIPTVHYNMGG
SDHA2_OVOL      EGRGVGKENDHIYLQLHHLPADHLHNKLPGIMETAQIFAGVDATKEPIPVVPTVHYNMGG
SDHA1_ASUU      EGRGVGPEKDHIYLQLHHLPAEQLHQRLPGISETAKIFAGVDVTKEPIPVIPTVHYNMGG
SDHA2_CELE      EGRGVGPNKDHIYLQLHHLPAEQLQQRLPGISETAQIFAGVDVTKEPIPVIPTVHYNMGG
SDHA_NAME       EGRGVGPEKDHIFLQLHHLPAKQLHERLPGISETAMIFADVDVTKEPIPVIPTVHYCMGG
SDHA_HCON       EGRGVGPEKDHIFLQLHHLPAQQLHERLPGISESAMIFANVDVTKEPIPVIPTVHYCMGG
SDHA1_BMAL      EGRGVGKDKDHIYLQLHHLPAKDLHAKLPGIMETAMIFAGVDAAKEPIPVIPTVHYNMGG
SDHA1_OVOL      EGRGVGKDKDHIYLQLHHLPAKDLHARLPGIMETAMIFAGVDAAEEPIPVLPTVHYNMGG
SDHA_SRAT       EGRGVGPEKDHIYLQLHHLPAEHLAQRLPGISETAKIFAGVDVTKEPIPVLPTVHYNMGG
SDHA_MHAP       EGRGVGPEKDHIFLQLHHLPAEQLNKRLPGISETAKIFAGVDVTKEPIPVLPTVHYNMGG
SDHA1_CELE      EGRGVGPDKDHIYLQLHHLPAEQLQQRLPGISETAMIFAGVDVTKEPIPVIPTVHYNMGG
SDHA2_ASUU      EGRGVGPEKDHIYLQLHHLPVEQLLTRLPGISETAKIFAGVDVAKEPIPVIPTVHYNMGG
                ******  :** :*:* *:* . :  .**** *:* :**.** : :*:**:***** ***

SDHA_TMUR       IPTNHMAQALTLKKGGG-DQIVEGLYAAGETAAHSVHGANRLGANSLLDLVIFGRACAVN
SDHA_SMED       IPTNYKGQVITYDEHNKKDKIVKGLYAAGEAASTSVHGANRLGANSLLDIVVFGRACALT
SDHA_EGRA       VPTNYKGQVITYDAATGKDKIIPGLYAAGEVACASVHGANRLGANSLLDLVVFGRACALD
SDHA_EMUL       VPTNYKGQVITYDAATGKDKIIPGLYAAGEVACASVHGANRLGANSLLDLVVFGRACALD
SDHA_HMIC       VPTNYKGQVITYDPVSMKDKVVPGLYAAGEVACASVHGANRLGANSLLDIVVFGRACALD
SDHA_FHEP       VPTNYKGQVLTYDPVAKKDKLIPGLYAAGEAACASVHGANRLGANSLLDLVVFGRACALD
SDHA_SMAN       IPTNYKGQVLAYDPVAKKGKVVPGLYAAGEAASASVHGANRLGANSLLDIVVFGRACALD
SDHA2_BMAL      IPTDYKGQVITFSHSEG-DRLVPGLFACGETAAHSVHGANRLGANSLLDTVVFGRACANN
SDHA2_OVOL      IPTDYKGQVITFSPSKG-DQLVPGLFACGETAAHSVHGANRLGANSLLDTVVFGRTCANN
SDHA1_ASUU      IPTNYKAQVIKYTKEGG-DKIVPGLYACGECACHSVHGANRLGANSLLDAVVFGRACSIN
SDHA2_CELE      VPTNYKGQVLDFTPEGG-DKVIPGLYAAGECAAHSVHGANRLGANSLLDLVIFGRSCALT
SDHA_NAME       VPTNYKGQVISYSKEKG-DQIIPGLYAAGECSAQSVHGANRLGANSLLDLVIFGRAAALS
SDHA_HCON       IPTNYKGQVLSYSKDKG-DQVVPGLYAAGECSVQSVHGANRLGANSLLDLVIFGRSCALT
SDHA1_BMAL      IPTNYMGQVLTYKRDKG-DQLVPGLYACGEAAAHSVHGANRLGANSLLDLVVFGRACAID
SDHA1_OVOL      IPTNYMGQVLTHKPDRG-DQVVPGLYACGEAAAHSVHGANRLGANSLLDLVVFGRACAID
SDHA_SRAT       TPTNYKGQVITYTEEGG-DKIVPGLYAAGECAAHSVHGANRLGANSLLDLVIFGRACALD
SDHA_MHAP       TPTNYKGQVIKYTRDKG-DQIVAGLWAAGECAAHSVHGANRLGANSLLDLVIFGRACALN
SDHA1_CELE      VPTNYKGQVLNYTPKKG-DEVVPGLYAAGECGAHSVHGANRLGANSLLDLVIFGRACAID
SDHA2_ASUU      VPTNYMGQVLKYTRAKG-DQLVPGLYAAGEAAAHSVHGANRLGANSLLDLVIFGRACALS
                 **:: .*.:        . :: **:*.** .  *************** *:***:.:

SDHA_TMUR       IAKLCK-PGEKIPDMPSDAGEASVANLDKVRNAK--GSISVADLRLKMQKTMQEHASVFR
SDHA_SMED       IAEENK-PGETQPDLAKDAGEASIANLDKVRNAN--GTLSTAEIRLKMQKTMQDHAAVFR
```

```
SDHA_EGRA       IAEKCK-PGDFGPELHPETGMESVANIDKLRSGK--GQFPVSDVRLEMQRTMQEHAAVFR
SDHA_EMUL       IAEKCK-PGDFGPELHPETGMESVANIDKLRSGK--GQFPVSDVRLEMQRTMQEHAAVFR
SDHA_HMIC       IAEKCK-PGDAGPELHPETGMESVANIDKLRSGR--GEFPVGDVRLEMQHTMQEHAAVFR
SDHA_FHEP       IAEKHK-PGDPGPELKPDAGEASITNLDKLRHAN--GTHPIADVRLEMQRTMQDYAAVFR
SDHA_SMAN       IAAKNK-PGDAGPELKPDTGEASIANYEKLRTAN--GSYPIAQVRLDMQRTMQEYAAVFR
SDHA2_BMAL      ILEQAKLTNFKLPDLPPNAGEATLANVDKMRFAK--GDIYTGALRLKMQKTMQKHAAVFR
SDHA2_OVOL      ILEQAKSFGLKVPDLLPNAGEASLANVDKMRFAN--GDIRTAALRLKMQKTMQKYAAVFR
SDHA1_ASUU      IKEELK-PDEKIPELPEGAGEESIANLDAVRYAN--GDVPTAELRLTMQKTMQKHAGVFR
SDHA2_CELE      ILNENK-PGDSIPELPVNCEEKSCDNLNGLLHSK--GDISSIELRQKMQMTMQKHAAVFR
SDHA_NAME       ILEHTK-PGVGVPDLPKDAGESSVDNIDSLMNRK--DGPSTADVRLQLQKTMQKHAAVFR
SDHA_HCON       ILGHTK-PGVGVPDLPKDAGEASVDNIDTLMHRK--DGPSTAKTRLELQKTMQKHAAVFR
SDHA1_BMAL      ILEKAKKSCEKIPELPKDAGESTITNVDKLRFAK--GDIPTAALRLKMQKTMQQHAAVFR
SDHA1_OVOL      ILKKAKESSEKIPELPKGAGESTIANVDKLRFAK--GDIPTAALRLKMQKTMQQHAAVFR
SDHA_SRAT       ILSKNK-PGDSIKPLPKNAGEETIQRVDSARHAN--GDISTAELRLNMQKTMQGHAAVFR
SDHA_MHAP       ILAQNK-SGETVPNLPPNAGEASIANVDRLRNGSKGAGVPTAQLRLEMQRTMQKHAAVFR
SDHA1_CELE      ILKNTS-AGVGVPELPKNAGEASVANIDKLRHNK--GDISTAELRLTMQKSMQNHAAVFR
SDHA2_ASUU      ILKNTK-PGETPPDLPANAGEASIANLDKMRHAN--GDIPTAELRLQMQKTMQKHAAVFR
                *    .        :       :  . :                *  :* :** :*.***

SDHA_TMUR       TGSIL-----------------EDGCKKMQDVYK-QIFDIK-LSDRSMIWNTDLMEALEL
SDHA_SMED       DGPVL-----------------KEGCEKVLEIYRNDMPQLK-LFDRSMVWNSDLVEALEL
SDHA_EGRA       DGPVL-----------------KAGVEKMLKLYAAKYDNLK-VSDKSLIWNSDLIEGLEL
SDHA_EMUL       DGPVL-----------------KAGVEKMLKLYAAKYDNLK-VSDKSLIWNSDLIEGLEL
SDHA_HMIC       DGPVL-----------------KAGVEKMLKLYADKYDNLK-VSDKSLIWNSDLIEGIEL
SDHA_FHEP       DGPTL-----------------QEGCKRMYDLYKSRMFDLK-VTDRSLIWNSDLIEALEL
SDHA_SMAN       DGPTL-----------------KEGCKKMYDLYASRMNDLK-VSDRSKIWNSDLMEALEL
SDHA2_BMAL      RGDIL-----------------QEGIKKMESIFG-EQKHLK-TLDRGLIWNSDLIETLEL
SDHA2_OVOL      RGDFL-----------------QEGIKKMESIYG-ELKHLN-TSDRGLIWNSDLIETLEL
SDHA1_ASUU      RGDIL-----------------AEGVKKMMDLFK-ELKRLK-TTDRSLIWNSDLTESLEL
SDHA2_CELE      RGDLL-----------------KEGVDKMSSIYK-EQQNLKACADSGKVWNSELVETLEL
SDHA_NAME       RGDILQARDAALPSNTNFSYYFQEGVKKVGEIYK-NIDNLH-VSDKSLIWNSDLVETLEL
SDHA_HCON       RGDIL-----------------QEGVKKVEDIYK-NYHNLH-VTDTSLIWNTDLIETLEL
SDHA1_BMAL      RGDIL-----------------KEGIKKMETLFK-EQKLLK-TTDRGLVWNSDLAETFEL
SDHA1_OVOL      RGDIL-----------------KEGITKMETLFK-EQKLLK-TTDRGLVWNSDLAETLEL
SDHA_SRAT       RGDIL-----------------KKGCDKIAELYK-QQKNIK-VTDRGLIWNSDLIETLEL
SDHA_MHAP       TGPIL-----------------QKGIEKIMNLYS-QLKHVH-ISDRTLIWNSDLIETLEL
SDHA1_CELE      RGDIL-----------------KEGVKVLSKLYK-DQAHLN-VADKGLVWNSDLIETLEL
SDHA2_ASUU      RGDIL-----------------AEGVEKMKGLYK-DLKHLK-TTDRGLVWNSDLIETLEL
                 *  *                   *   :  ::      ::   *   :**::* * :**

SDHA_TMUR       QNLLLNAVQTIESASARKESRGAHAREDFKQRIDEYDYSKPLEGQTKLPFEKHWRKHSLV
SDHA_SMED       QNLMLNSLQTIVGAENRKESRGAHSREDFKIRLDEFDYSKPVEGQIKKPVEQHWRKHTLA
SDHA_EGRA       QNLLINAVQTIVAAEARKESRGAHAREDFRQRIDEYDYSKPIEGQTKKPMSEHWRKHTMS
SDHA_EMUL       QNLLINAVQTIVAAEARKESRGAHAREDFRQRIDEYDYSKPIEGQTKKPMSEHWRKHTMS
SDHA_HMIC       QNLLINAVQTIVGAEARKESRGAHAREDFRQRVDEYDYSKPLEGQTKKPFADHWRKHTMS
SDHA_FHEP       QNLMLNALQTIVSAEARKESRGAHAREDFPNRLDEIDYSKPPEGQVRKPFSDHWRKHTLS
SDHA_SMAN       QNLMLNALQTIVGAEARKESRGAHAREDFPNRVDELDYSKPIDGQKPKPFEEHWRKHTLS
SDHA2_BMAL      QNLLLCAMQTISAAEARKESRGAHARDDFKQRIDEFDYSIPLAGQIKKPFKEHWRKHTVI
SDHA2_OVOL      QNLLLCAMQTIIAAEARKESRGAHARDDFKQRIDEFDYSIPLAGQTKKPMEQHWRKHTII
SDHA1_ASUU      QNLMLNATQTIVAAENRKESRGAHARDDFPKREDEYDYSKPIEGQTKRPFEKHWRKHTLT
SDHA2_CELE      QNLLINANQTIVAAENRTESRGAHARDDFQERIDEYDYSNPLEGQQKKPFDQHWRKHSII
SDHA_NAME       QNLLINAIQTIVAAEARKESRGAHARDDFPTRIDEYDYGKPLDGQKKKPIEEHWRKHTMI
SDHA_HCON       QNLLISALQTVVAAEARKESRGAHARDDFPTRIDEYDYSKPLEGQKKKPIEEHWRKHTMI
SDHA1_BMAL      QNLMLNATQTIIAAEARKESRGAHARDDFPTRIDELDYSRPLDGQTKKSLDQHWRKHTII
SDHA1_OVOL      QNLMLNATQTIVAAEARKESRGAHARDDFPTRIDEFDYSRPLDGQTKKSLDQHWRKHTMI
SDHA_SRAT       QNLLINAQQTIVAAENRKESRGAHARDDYPTRLDEMDYSKDTSNQTPKPITEHWRKHSII
SDHA_MHAP       QNLLANAMQTIVAAENRKESRGAHARDDYPNRSDEFDYSKPLEGQKKKTHEEHWRKHSII
```

```
SDHA1_CELE      QNLLINATQTIVAAENREESRGAHARDDFPDRLDELDYSKPLEGQTKKELKDHWRKHSII
SDHA2_ASUU      QNLMLNAMQTIVAAENRKESRGAHARDDFPNRIDEYDYSKPLEGQVKKPIDQHWRKHSII
                ***:  : **: .*. * ******:*:*:  * ** **.    .*      .*****::

SDHA_TMUR       WMDPATGKTRIDYRPVIDATLNKSEVDTVPPKVRSY
SDHA_SMED       SQNVATGDVKLSYRPVIDKTLDDSKCHTVPPQIRSY
SDHA_EGRA       YQNLKTGAVKLEYRPVIDKTLDQNVCPTVPPKIRSY
SDHA_EMUL       YQNLKTGAVKLEYRPVIDKTLDQNVCPTVPPKIRSY
SDHA_HMIC       YQDIKTGAVKLEYRPVIDKTLNENVCPSVPPKIRSY
SDHA_FHEP       FQDVKTGKVTLDYRPVIDATLDAKQCPTVPPKVRSY
SDHA_SMAN       YQDVKTGAVKLEYRPVIDATLDAKSCPSVPPKLRTY
SDHA2_BMAL      SQNPETGKVALSYRPVIDQTLDENEVEPIPPIIRKY
SDHA2_OVOL      SQNPETGKVSLSYRPVIDQTLDENEIKTIPPIIRKY
SDHA1_ASUU      KQDPRTGHITLDYRPVIDKTLDPAEVDWIPPIIRSY
SDHA2_CELE      GIDTKTGAVDLTYRPVIDKTLDKSETDWVPPKVRSY
SDHA_NAME       TSDIKTGKVKLDYR----------------------
SDHA_HCON       TTDIKTGKVKLEYRPVIDTTMDKNEVDWIPPK----
SDHA1_BMAL      EQDHETGKITLHYRPVIDQTLDKNETDWVQPMIRSY
SDHA1_OVOL      EQNHETGKITLLYRPVIDQTLDKSETDWIQPMIRSY
SDHA_SRAT       TQNTETGDVKLAYRPVIDITLDKSETDWVPPKVRSY
SDHA_MHAP       YQDPETGKVTLEYRPVIDKTLDKTE-----------
SDHA1_CELE      RSNIETGEVSLDYRPVIDTTLDKSETDWVPPKVRSY
SDHA2_ASUU      YQEPETGKVTLDYRPVIDKTLDKSETDWVPPKVRSY
                  :  **   : **
```

SDHB

```
SDHB2_EGRA      ----------------------------------------NPDKPDQKPYMQTFEIDTSD
SDHB2_EMUL      ----------------------------------------NPDKPDQKPHMQTFEVDTSD
SDHB_HMIC       ----------------------------------------NPDKPEQKPYMQAFTIDTSD
SDHB1_EGRA      ----------------------------------------NPDKPDQKPYMQTFEVDTSD
SDHB1_EMUL      -------------------------ASTGPVMKKFSIYR----------YMQTFEVDTSD
SDHB_SMED       ----------------------------------------NPDKPKEKPKMDTYEIDLNK
SDHB_FHEP       ----------------------------------------NPDKPGEKPRMQDYKVDLND
SDHB_SMAN       ----------------------------------------NPDKRGEKPTMQNYQVDLND
SDHB_TMUR       ----------------------------------------NPDTPAVKPFVQTYKIDLKQ
SDHB_MHAP       --------------------AVSVASVQSSNVKTFEVYRYNPETPEVKPYLQKYDIDLNK
SDHB_BMAL       --------------------------------KTFEIYRYNPEKPGSQPQLQKYDIDLDD
SDHB_OVOL       --------------------------------KTFEIYRYDPEKPGSQPKLQKFDIDLDD
SDHB_SRAT       -------------------------CSKGANIKTFEIYRYNPDKPGSKPEVKKYDVDLNK
SDHB1_HCON      --------------------------------KTFEIYRWNPEKKGSKPEMQKYEVDLDD
SDHB_ASUU       --------------------------------KTFEIYRFNPEEPGAKPKLQKFDVDLDK
SDHB2_HCON      --------------------------------KTFEIYRWNPDTPSVKPSLKKYDVDLDD
SDHB_CELE       ASGAPATAAAAEASFPSTDDVAAKTKKTGNRIKTFEIYRFNPEAPGAKPTVQKFDVDLDQ
SDHB_NAME       ------------------------------------------------------------

SDHB2_EGRA      CGPMVLDALIKIKAECDPALTFRRSCREGICGSCAMNIDGRNHLACIYAIPKN-NKITKI
SDHB2_EMUL      CGPMVLDALIKIKAECDPTLTFRRSCREGICGSCAMNIDGRNHLACIYAIPRS-NKVTKI
SDHB_HMIC       CGPMVLDALIKIKTECDPTLTFRRSCREGICGSCSMNIAGRNRLACLCAIPKD-NKVTKI
SDHB1_EGRA      CGPMMLDALIKIKSDQDPTLTFRRSCREGICGSCSMNINGRNRLACLCAIPKD-NKVTKI
SDHB1_EMUL      CGPMMLDALIKIKSDQDPTLTFRRSCREGICGSCSMNINGRNRLACLCAIPKD-NKVTKI
SDHB_SMED       CGPMVLDALIKIKNEMDPTLTFRRSCREGICGSCAMNIGGLNTLACICNIDKNIGKTTKI
SDHB_FHEP       CGPMVLDALIKIKNEQDPTLTFRRSCREGICGSCAMNIGGRNHLACIWEIDKNLSKPTKI
SDHB_SMAN       CGPMVLDALIKIKNEQDPTLTFRRSCREGICGSCAMNIEGRNHLACIWEIDPDINKTTKI
SDHB_TMUR       CGPMVLDALIKIKNEQDATLTFRRSCREGICGSCAMNINGQNTLACTCKIDDSINKPEKI
SDHB_MHAP       CGPMVLDVLIKIKNEIDPTLTFRRSCREGICGSCSMNINGENTLACICHIDKITNKSTKI
```

```
SDHB_BMAL       CGAMILDALIKIKNEIDPTLTFRRSCREGICGSCAMNINGENTLACICKIDENTSKSTKI
SDHB_OVOL       CGAMILDALIKIKNEVDPTLTFRRSCREGICGSCAMNIDGQNTLACICKIDGNTSKSSKI
SDHB_SRAT       CGPMVLDALIKIKNEQDPSLTFRRSCREGICGSCAMNIGGANTLACICNIDKNTSKSTKI
SDHB1_HCON      CGSMVLDALLKIKSEQDPTFTFRRSCREGICGSCAMNIAGENNLACTLKIDTDLSKTTKI
SDHB_ASUU       CGTMVLDALIKIKNEVDPTLTFRRSCREGICGSCAMNIAGENTLACICNIDQNTSKTTKI
SDHB2_HCON      CGSMVLDALNKIKDEQDPTLTFRRSCREGICGSCAMNIDGENTLACIKKINTDTSKSTKI
SDHB_CELE       CGTMILDALIKIKNEVDPTLTFRRSCREGICGSCAMNIGGQNTLACICKIDSDTSKSTKI
SDHB_NAME       ---MVLDALIKIKNEVDPTLTFRRSCREGICGSCAMNIGGENTLACIQKIDTDTSKTTKI
                   *:**.* *** : *.::**************:*** * * ***   *    .*  **

SDHB2_EGRA      YPLPHSYVVKDLVVDIANFYSQFRWIEPYLKKKEFTDEQIGEKFFLQSLKDSKKLDNLQA
SDHB2_EMUL      YPLPHSYVVKDLVVDIANFYSQFRWIEPYLKKKEFTDDQIGEKFFLQSLKDSKKLDNLQA
SDHB_HMIC       YPLPHMYVVKDLVPDMANFYAQYRWIEPYLKKKEFTDEQIGEKVFMQSVKDREKVDGLYE
SDHB1_EGRA      YPLPHMYVVKDLVPDMANFYAQYRWIEPYLKKKEFSEEQVGEKVFMQSIKDREKIDGLYE
SDHB1_EMUL      YPLPHMYVVKDLVPDMANFYAQYRWIEPYLKKKEFSEEQVGEKVFMQSVKDREKIDGLYE
SDHB_SMED       YPLPHMHVIKDLVPDMSNFYAQYRWIEPYLKKKSENEADIGKKVNYQSVKDRDVLDGLYE
SDHB_FHEP       YPLPHMFVIKDLVPDMNNFYAQYRSIEPYLKKKNVREEDLGKATYYQSVEDRSKLDGLYE
SDHB_SMAN       YPLPHMYVIKDLIPDMNNFYAQYRFIEPYLKKKNVDEEDIGKKTYYQSVEDRAKLDGLYE
SDHB_TMUR       YPLPHMYVIKDLVPDLSLFYQQYRSIDPWLKRK--DSFKLGEKQFYQTIEERARLDGLYE
SDHB_MHAP       YPLPHMYVVKDLIPDMTLFYQQYGSIQPWLQKN--KPLKLGEKEMYQTIDERATLDGLYE
SDHB_BMAL       YPLPHMFVIKDLVPDMNLFYAQYASIEPWLKKK--NKLVLGEKQMFQTEKEREKLNGLYE
SDHB_OVOL       YPLPHMFVIKDLVPDMNLFYAQYASIEPWLKKK--NKLILGEKQMYQTEKEREKLDGLYE
SDHB_SRAT       YPLPHMYVVKDLVPDMNLFYAQYSSIQPWIQRN--TPIKLGDKQLYQSVDEREKLDGLYE
SDHB1_HCON      YPLPHMYVIKDLVPDLSLFFEQYRSIQPWLQKN--EKLTLGEKQMFQSADERARIDGLYE
SDHB_ASUU       YPLPHMFVIKDLVPDMNLFYAQYASIQPWLQKK--TKINLGEKQQYQSIKEQEKLDGLYE
SDHB2_HCON      HPLPHMYVMKDLVPDLTLFFEQYRSIQPWLQKK--TPLKLGDKQMFQSEKERERLDMLYE
SDHB_CELE       YPLPHMFVVKDLVPDMNLFYAQYASIQPWIQKK--TPLTLGEKQMHQSVAERDRLDGLYE
SDHB_NAME       YPLPHMFVVKDLVPDMNLFYEQYASIQPWLQKK--TSLKLGDKQMFQSVKERDRLDGLYE
                :**** .*:***: *:  *: *:  *:*:::.:      :*.    *:  :   :: *

SDHB2_EGRA      CILCFCCSAACPSYWWNSDKYLGPATLLHAYRWIVDSRDDFTFERLEQLQNKWSVYRCHT
SDHB2_EMUL      CILCFCCSAACPSYWWNSDKYLGPATLIHAYRWIVDSRDDFTFERLEQLQNKWSVYRCHT
SDHB_HMIC       CILCLCCSASCPSYWWNSDKYLGPAVLQQAYRWMIDSRDDYTFDRLEQMQNKWSTYRCHT
SDHB1_EGRA      CILCLCCSASCPSYWWNSDKYLGPAVLQQAYRWMIDSRDDYTFDRLEQMQNKWSTYRCHT
SDHB1_EMUL      CILCLCCSASCPSYWWNSDKYLGPAVLQQAYRWMIDSRDDYTFDRLEQMQNKWSTYRCHT
SDHB_SMED       CILCACCSTSCPSYWWNGDKYLGPAVLMQAYRWMIDSRDEFTYERLQQMNNGWSAYRCHT
SDHB_FHEP       CILCACCSASCPSYWWNQDKYLGPAVLLQAYRWLIDSRDDYTYERLTNFQNKWSMYRCHT
SDHB_SMAN       CILCACCSTSCPSYWWNGDKYLGPAVLLQAYRWLVDSRDDYTYERLAEFQNKWSLYRCHT
SDHB_TMUR       CILCACCSTSCPSYWWNADKYLGPAILLQTYRWLIDSRDHYTKERLERIQDGFSAFRCHT
SDHB_MHAP       CILCACCSTSCPSYWWNPDKYLGPAALLQSYRWIIDSRDDKAKERLSRLTDPFSAFKCHT
SDHB_BMAL       CILCACCSTSCPSYWWNADKYLGPAILLQSYRWMIDSRDDYAEERLSKIHDHFSVFKCHT
SDHB_OVOL       CILCACCSTSCPSYWWNADKYLGPAILLQSYRWMIDSRDDFAEERLSKIHDHFSAFKCHT
SDHB_SRAT       CILCACCSTSCPSYWWNSDKYLGPAVLMQAYRWIIDSRDDKHNERLDRMRDAFSAFKCHT
SDHB1_HCON      CILCACCSSSCPSYWWNADKYLGPAVLMQAYRWIVDSRDDYARERLARIHDAYSAFKCHT
SDHB_ASUU       CILCACCSASCPSYWWNADKYLGPAVLMQAYRWIIDSRDDSAAERLARMQDGFSAFKCHT
SDHB2_HCON      CILCACCSSSCPSYWWNADKYLGPAVLMQAYRWIIDSRDDYPKERLARMHDAFSAFKCHT
SDHB_CELE       CILCACCSTSCPSYWWNADKYLGPAVLMQAYRWVIDSRDDYATERLHRMHDSFSAFKCHT
SDHB_NAME       CILCACCSTSCPSYWWNADKYLGPAVLMQAYRWIIDSRDDYPMERLHRMHDAYSAFKCHT
                **** ***::******* ******* * ::***::****    :**  : : :* :.***

SDHB2_EGRA      IMNCTQTCPKGLNPGRAIAEIKKMLIYYSSYKNKK-PMNDRLENADPT
SDHB2_EMUL      IMNCTQTCPKGLNPGRAIAEIKKMLIYYSSYKNKK-PMNDRLENADPT
SDHB_HMIC       IMNCTETCPKGLNPGKAIGEIKKMLIYYNSYKNKK-PMNERLENSVPV
SDHB1_EGRA      IMNCTETCPKGLNPGKAIGEIKKMLIHYNSYKNKK-PMNERLENSVPF
SDHB1_EMUL      IMNCTETCPKGLNPGKAIGEIKKMLIHYNSYKNRK-PMNERLENSVPF
SDHB_SMED       IMNCTKTCPKGLNPGLAIGKIKVMLMNLNSYK----RDEVKA------
SDHB_FHEP       IMNCTETCPKELNPGLAIGEIKKMLIYYNQYKDKP-PMTKTV------
```

```
SDHB_SMAN       IMNCTETCPKGLNPGLAIGEIKKMLIYFNQYKHHK-PETRTV------
SDHB_TMUR       ILNCTKACPKYLDPAKAIANIKQLLSGFKKKPEPV-PEPQTVPGSNV-
SDHB_MHAP       ILNCTKTCPKHLNPAKAIGEIKSMLTGLRTKPEPQVPKPADS------
SDHB_BMAL       ILNCTKTCPKHLNPAKAIGEIKKLLTGFDKKPAPV-AAPANF------
SDHB_OVOL       ILNCTKTCPKHLNPAKAIGEIKKLLTGFDKKPAPM-AAPANF------
SDHB_SRAT       ILNCTKTCPKHLNPAQAIGEIKKLLTGFGKKPAPH-PAPADF------
SDHB1_HCON      IMNCTKTCPKHLNPARAIGEIKSLLTGMKNKPA---PEPAKF------
SDHB_ASUU       IMNCTKTCPKHLNPARAIGEIKMLLTKMKTKPAPL-PTPANF------
SDHB2_HCON      IMNCTKTCPKNLNPAKAIGEIKTLLTGFKSKPT---PEPAKF------
SDHB_CELE       IMNCTKTCPKHLNPAKAIGEIKSLLTGFTSKPA---AEPSAF------
SDHB_NAME       IMNCTKTCPKHLNPAKAIGEIKSLLTGIKTKPA---PEPAKF------
                *:***::*** *:*. **.:** :*
```

SDHC

```
SDHC_SRAT       --------------------------------------------DSKTPIQKFGWNYLQT
SDHC_TMUR       ------------------------SAVAAHRCALGLKTFAPLSSDVKTPIQAWGWDYLQK
SDHC_MHAP       ---------------MTIISIIPSVLKFRNLLPNKCGRSIRLTHTSKTPVQQFGFEYLKQ
SDHC_ASUU       -------------------------------------------SAEKTPIQVWGWDYLMR
SDHC_BMAL       -------------MLFQMKNAAFCRALSQNVQTLRLLSTSVPQQSIKTPIQEWGWNYLLR
SDHC_OVOL       -------------------------------QVLRLLKTGVPQQSVKTPIQEWGWDYLLR
SDHC_CELE       -------------------------------------------SEAKTPIQKFGWEYLLK
SDHC_NAME       --------------------------------------------DVKTPIQEWGWAYLMR
SDHC_HCON       --------------------------------------------DVKTPIQEWGWTYLQR
SDHC_SMED       MSMILKGIRMGTFGARLLRNPQVFYPVRTQYLKYCCGTSEKVRLRAQTEMQDY-WE---K
SDHC_SMAN       ------------------------SSIVRTSKAAKGSTSEKVRRQAEKEMQSF-WD---R
SDHC_FHEP       --------------------------------------------QAQKEMQAF-WE---R
SDHC1_HMIC      ------------------------TPVLNITANCKGTSPEDVRLQAQTEMQDY-WE---K
SDHC2_HMIC      ------------------------SPVLTLTSYGKGSTSDEVRHLAQSEMQDY-WE---R
SDHC_EGRA       ------------------------APVLSATKHYKGSTSEEVRLKAESEMQDY-WE---R
SDHC_EMUL       ------------------------APVLSATKHYKGSTSEEVRLKAESEMQDY-WE---R
                                                              :. :* : :

SDHC_SRAT       QKALKRPLSPSIGIYQMQMTWGLSGLSRISGSIMGGVLVVGGAGFALLPYNFTQFCELIE
SDHC_TMUR       QKELHRPIAPHLTVYKPMLTWMLSGLHRITGVAMGTTIAIFSVGLMAVPFDFTAVVSFIR
SDHC_MHAP       QTSLGRPISPHLNIYKPQLTWIISGGHRISGCIMSGTLLFGVLAFSFGPFNYSLFIERIR
SDHC_ASUU       QRALKRPIAPHLTIYKPQMTWMVSGLHRVTGCAMAGTLLIGGVGFSVLPLDFTTFVEFIR
SDHC_BMAL       QRALKRPISPHLAVYKPQVTWMVSGFHRMTGCAMAGTLLIGGVGFALLPLNFTTFIDFIR
SDHC_OVOL       QRALKRPISPHLSVYKPQLTWMVSGFHRMTGCAMAGTLLIGGVGFALLPLNFTTFIEFIR
SDHC_CELE       QRSKNRPIAPHLTVYQPQLTWMLSGFHRISGCVMAGTLLVGGIGFAVLPFDFTAFVDFIR
SDHC_NAME       QKKLGRPISPHLTVYQPQLTWMVSGLHRVTGCVMAGVLLFGSIGFAALPFNFTQFVEYIR
SDHC_HCON       QKALGRPIAPHLTVYKPQLTWMVSGFHRVTGCAMAGTLLLGGVGFALAPFNFTQFVDYIR
SDHC_SMED       NLKGPRPYSPHLLIYKMELPMTTSLLHRGTGIAMAIAWGGLGVGAFWYTGQYAELINYVS
SDHC_SMAN       NATEKRPWSPHLQVYSSPLVMRFSFLHRATGIAMAIVWSSVGIGAFFFTGHYDSILDYVR
SDHC_FHEP       NIQEKRPWSPHLSVYSPPLVMRFSFLHRATGIAMAFVWMGVGCSAFLFTGHYEAMLDYVR
SDHC1_HMIC      NLKLKRPLSPHATIYSPPLCMATSFMHRATGVVMALAWMSAGCAGFWYTGNFDAMLEYVN
SDHC2_HMIC      NIKLKRPVSPHATIYKPPLCMCTSFMHRSTGVVMCLAWMALGCGGFWYTGNFDAMLEYIN
SDHC_EGRA       NIKLKRPWSPHILIYSPPLCMRNSFLHRATGVAMAIVWMGAGAAGFWYTGHFDGMLDYVS
SDHC_EMUL       NIKLKRPWSPHILIYSPPLCTRNSFLHRATGVAMAIVWMGAGAAGFWYTGHFDGMLDYVS
                :    ** :*   :*.  :    *   * :*  *  .      .    .  :  . . :

SDHC_SRAT       SFHIPSPILDAFKFVVAFPIVYHIFNGIRFLGYEFGIGAD-LATIYKSGYFVLIVSAAIA
SDHC_TMUR       DLQLPALLVYAVKYAIAWPLTYHTLNGVRFLGFDLAKGTD-IPTVYKTGWTVVGLSVIIA
SDHC_MHAP       SWNLPWPVTAIFKFIIAWTIVFHSLNGIRFMGFDLAKGME-LRQIYMSGYLVLGLSTLIS
SDHC_ASUU       GLGIPWVILDTFKFIIAFPIAFHTLNGIRFIGFDMAKGTD-IPSIYRGAYLVLGLAALIS
SDHC_BMAL       GLGLPWVITDIFKFIIAYPIAFHSLNGIRFIAFDLALGTD-IASVYTSGYLVLSLAALIA
SDHC_OVOL       GLGLPWVITDTLKFIIAYPIVFHSLNGIRFIAFDLALGTD-IASVYSSGYLVLSLAALIA
SDHC_CELE       SWNLPCAVTAVFKYIIAFPIIFHTLNGIRFLGFDLAKGVNNVGQIYKSGYLVSGLSAILA
```

```
SDHC_NAME       SWHIPPIITSVFKFVIAFPIIFHTLNGIRFIGFDLAKGVDNTKAIYKSGYLVLGLSAIIA
SDHC_HCON       SWHIPPVVTSVFKFVIAFPIVFHTLNGIRFIGFDLAKGVENTAAIYKSGYLVLGLSFIIA
SDHC_SMED       SLDLATPIIYSAKLIMCWPLIFHYTNGMRHLAYDAARGFD-IPTTMRTGYIAIAVSIVLA
SDHC_SMAN       NMHLGTSVIAACKFILCYPLVYHYLNGMRHLAWDYAIGFP-IKTCNTTGFIALGSSLVIS
SDHC_FHEP       NLHLGSLVITGCKFVLCYPLVYHYMNGMRHLAWDYAIGFP-IKTCNMTGMTVLSLSLVAA
SDHC1_HMIC      SFQFGSTVLYGAKFLLAYPLVYHYTNGMRHLAWDYAIGFD-MKTVNLTGSTNLMMSFVIT
SDHC2_HMIC      SYHFAPYYLFGAKFLLAYPLIYHYTNGIRHLAWDYSIGFD-MKTVNFTATTNLIFSFLLT
SDHC_EGRA       SFSFGPSIVFGAKCLLAYPLVYHYCNGMRHLAWDYAIGFD-MKTVNMTGATVLILSVLLT
SDHC_EMUL       SFSFGPSIVFGAKCLLAYPLVYHYCNGMRHLAWDYAIGFD-MKTVNMTGATVLILSFLLT
                .  :        *  :.:.: :*  **:*.:.:: . *          .      :   :

SDHC_SRAT       LFCVLNAQRRPIKEVLKN----
SDHC_TMUR       AAITF---WPKD----------
SDHC_MHAP       LLIVANSKRISAEEMEEMY---
SDHC_ASUU       LAVVVYPRWERHKKATLPTNH-
SDHC_BMAL       LAVVVAPRLKQEDYVVVDIPKK
SDHC_OVOL       LAVVVAPLLKKEDYVVVNEPKK
SDHC_CELE       LAIVFNSCQNKSNKTA------
SDHC_NAME       AAVVVNA-WPKQCCEEHTTKKH
SDHC_HCON       AAVVINA-WP------------
SDHC_SMED       TLFASVKL--------------
SDHC_SMAN       AILACIRL--------------
SDHC_FHEP       AALACVRL--------------
SDHC1_HMIC      ALLASIKL--------------
SDHC2_HMIC      LALASIKL--------------
SDHC_EGRA       LALASIRS--------------
SDHC_EMUL       LALASIRS--------------
                   .
```

SDHD

```
SDHD_HMIC       ------------------------------------------------------------
SDHD_EGRA       ------------------------------------------------------------
SDHD_EMUL       ------------------------------------------------------------
SDHD_SMED       ------------------------------------------------------------
SDHD_SMAN       -------------------------------------------SNKLGTAPA-------V
SDHD_SRAT       ------------------------------------------CSGVASKVPQ---FEALS
SDHD_MHAP       ------------------------MICGRFFQSAANPSIYKRLIKIGSVRPLCFSINNVV
SDHD_OVOL       -----------------------------------------YVAGGVSDRPQ---FDPIV
SDHD_BMAL       MERLFAGVHELADGVMGLMLSATGRIVPVSTRFVYPRLLQRSLAEVVSDRPQ---FDPIV
SDHD1_ASUU      ----------------------------------------AGATSAAVTGAAPPQFDPIA
SDHD2_ASUU      ----------------------------------------TSVTTPVSREPF--------
SDHD_CELE       -------------------------------------------TSTLNDGAS--------
SDHD1_NAME      -------------------------------------------ASTLDDGAN--------
SDHD_HCON       ------------------------------------------------------------

SDHD_HMIC       -----------------------------------YPIALIYEPYGFEHLVTLSVTLHAY
SDHD_EGRA       ---------------------------VALSMLPMYPIAIIYEPYLMDYVVSAAVSLHAY
SDHD_EMUL       ---------------------------VALSMLPMYPIAIIYEPYLMDYVVSAAVSLHAY
SDHD_SMED       ---------------------------VAVSMLPIYPAAWILENQFMSLLVVTTTSRTLI
SDHD_SMAN       QRTG-------LLP----SYHWTFERILAASMLPLYPIALYMDTPMMNLIVVTAVSMHSY
SDHD_SRAT       NKPL-------PVDMKPAK-EFLIEKYLTVAFMPFIPAAYFIHGPAMDMALAIGCSIHVY
SDHD_MHAP       ASKMNSSSTAQSAAMGPYALQFKLERYLAAGMFPLLPAAYFIHGTTMDLLLSAAIVMHSH
SDHD_OVOL       ARKT-------FKETHNHSMMFKIEKYFSAAMLPLLPAAYFIHGPTMDTALAIAITLHVH
SDHD_BMAL       ARKK-------FKETHNHSTMFKIEKYFSAAMLPLLPAAYFIHGPVMDTALAVAITLHVH
SDHD1_ASUU      AEKG-------FKPLHSHGTLFKIERYFAAAMVPLIPAAYFIHGREMDLCLALALTLHVH
SDHD2_ASUU      -------------SIEDHSLHFKIERYWAAGMIPLIPTAYFIHTPAMDAVLTVAIVLHVH
```

```
SDHD_CELE       -------------KVPDHSMHFKLERLWAVGMLPILPASYFIHGPVMDAVLTVALTLHIH
SDHD1_NAME      -------------KTHDHSLHFKLERYFAAGMVPLIPAAYFIHGPVMDAVLTVALTLHIH
SDHD_HCON       -----------ASKTHDHSLHFKIERYFAAAMVPLFPAAYFIHGPVMDAVLTVALTLHIH
                                                    * :       :.  :

SDHD_HMIC       -----WGFGGVLRDYLMERRYGAVLPKVMQ-ILWKVICLCGFAGFTYFNIYDIGVIKGVK
SDHD_EGRA       -----WGFGGVIRDYAIERKYGPLVPKVLQ-LLWKAICLFGFAGFTYFNYYDIGFIKGVK
SDHD_EMUL       -----WGFGGVIRDYAIERKYGPLVPKVLQ-LLWKAICLFGFAGFTYFNYYDIGVIKGVK
SDHD_SMED       FDLRFRGLDGVLKDYVMERRYGPYLQPIMR-NAWKVISAIGFAGFLYFEFNDIGFVKAVK
SDHD_SMAN       -----WGFDGVIKDYAFERRYGPALMPILR-TLWKVMAGFGFAGLLYFNFNDIGFISAVK
SDHD_SRAT       -----LGWHMVTTDYARPFLYGNTLAKIGR-ASAIVFAITCLAGLLHFNYNDVGLTKAFE
SDHD_MHAP       -----WGLMSVVQDYARPIVVGERLARISP-ALVYVTSTILLVGLLHYNLFDIGLTKSFE
SDHD_OVOL       -----WGLHGVLSDYGRPYVIGPTMAKIVQGPLSYIISICLLAGLLHFNSYDVGITKAFE
SDHD_BMAL       -----WGLHGVLSDYGRAFVLGTTAAKIVQGPVSYILSICLLAGLLHFNSYDVGITKAFE
SDHD1_ASUU      -----WGVWGVVNDYGRPFVLGDTLAAAVR-VGAYIFTACLLAGLLYFNEHDVGLTRAFE
SDHD2_ASUU      -----WGIAGVVSDYARPFVIGDTLARVAR-ASVYIITVILLASLLHFNNSDVGLTKAFE
SDHD_CELE       -----WGIHGVVYDYARPYVIGEAAAKAAH-VGVYLITGLLLGALLHFNTNDVGITKAFE
SDHD1_NAME      -----WGVQGVIQDYARPFVIGDAAAKAAR-AGVYLITAALLAGMFSFRAHRI-ITK---
SDHD_HCON       -----WGVQGVVQDYARPFVIGDTLAKTAR-ACVYLITAAILAGLLHFNTNDVGLTEAFR
                      *   *  **      *                   : .:  :    : .

SDHD_HMIC       KLWTF
SDHD_EGRA       KIWSL
SDHD_EMUL       KIWSL
SDHD_SMED       KLWSI
SDHD_SMAN       KLWAL
SDHD_SRAT       MVFAL
SDHD_MHAP       KVFSL
SDHD_OVOL       MVWSL
SDHD_BMAL       MVWSL
SDHD1_ASUU      MVWEL
SDHD2_ASUU      MVWSL
SDHD_CELE       LVFSL
SDHD1_NAME      -----
SDHD_HCON       LVFAL
```

NDUF2

```
NDUF2.1_FHEP       ------------------------------------------------------------
NDUF2.2_FHEP       ------------------------------------------------------------
NDUF2_SMED         -------------FNLNEINMIGFKLTSFIIKNSNLRYDSYLNIFKRYYAARWRIDDEFY
NDUF2_SMAN         ------------------------------------------------------------
NDUF2_HMIC         --------------------------------------------------------KEF-
NDUF2_EGRA         -------------------------MFSLCRATSSVTSKNFIETVRRALSSRWMPDKAF-
NDUF2_EMUL         -------------------------MFSLSRATSSVTSKNFIETVRRALSSRWMPDKAF-
NDUF2_TMUR         -------------------------------------------------GPVWYPDGKFY
NDUF2.2_OVOL       -------------------------------------MLFSLVQNTRHSHALWFPDAKFQ
NDUF2.2_BMAL       MESVKVEDAITLTLFLADMDFVRSSSLSLLSLKLRSSMLPAVGQNVRHSHALWFPDAKFH
NDUF2.1_OVOL       -------------------------------------VPNTLSFGKRSSHDLWYPDAEFM
NDUF2.1_BMAL       -------------------------------------APSTLSFGKRGSHNLWFPDAEFM
NDUF2_MHAP         --------------------MQTARRLITQNTFLLESNVPLISFKNRRHLHIWFPDAKFS
NDUF2.2_ASUU       ------------------------------------SAMSVASQSKRDSHTIWYPDAKFE
NDUF2_SRAT         -------------------------------------TTILRNQQLRNGHTIWYPDAEFE
NDUF2.1_ASUU       ----------------------------------------------RSAHTIWYPDAKFE
NDUF2.1_CELE       -------------------------------NVPSVAATSSTPATQTRNSHTIWYPDAKFE
NDUF2.2_CELE       -------------------------MLSRSLHPLRAVACARPAISNRDSHTIWYPDAKFE
NDUF2_HCON         ------------------------------------AGASQALLAQRASHTIWYPDAKFE
NDUF2_NAME         ------------------------------------AGASQALLAQRSSHTIWYPDAKFE
```

```
NDUF2.1_FHEP        --------------------------------------------------MTVIKE----
NDUF2.2_FHEP        -------------------------------MELTYPLAGDSRVKEKIE-----------
NDUF2_SMED          ----------------------------KKREYVKLDTSLPELE---AK-KRAAYA----
NDUF2_SMAN          ------------------------------MDELKESLYNPAVVKANTTVQPIDFF----
NDUF2_HMIC          ----------------------------VDAQKSLYKLPKERMDPEYVK-RRNMFD----
NDUF2_EGRA          ----------------------------VDAQQSLYKTPIEKIDPAYVK-RTNMFE----
NDUF2_EMUL          ----------------------------VDAQQSLYKTPIEKIDPAYVK-RTNMFE----
NDUF2_TMUR          ARFHEAY-----------VEPEQYSDAFRPDEETLKHIRTDSVGRRENL-QP--------
NDUF2.2_OVOL        HEFKKAS-----------VLGHIWAP--KA-TEYEKQVGTSNILEEYCR-RPRISD--IT
NDUF2.2_BMAL        HEFKKAS-----------VIGRVWAA--KT-TEYEKHVGTSEILENYCR-RPRMSD--IA
NDUF2.1_OVOL        REFKKAS-----------VIGRVWMP--EP-TEYDKNVGIDKVFEKSSW-QPTLPD--LS
NDUF2.1_BMAL        HEFKKAS-----------VVGRVWMP--KP-TEYEKNVGIDQVFEKSSW-QPVVSD--LT
NDUF2_MHAP          RMFKEVGHTKSAHQSLLQETGAVERM--EPDDLANPKAVLDAFDRM----EKATFDGLFG
NDUF2.2_ASUU        REFKTAG-----------MLGKLWFT--RR-TEFDKQIGLDKFEKLMVS-SPVQSDDYKG
NDUF2_SRAT          RDFKTAG-----------TLGRVHFP--KIDDKFEKDIGLEKLQKLLLE-EPVFSDSFGG
NDUF2.1_ASUU        RQFKKGG-----------TLGTLLFS--ERTTRYDEQLGMDKFEKLMEN-QPIQSDDYAG
NDUF2.1_CELE        RQFKTGG-----------TLGKLWMS--ERVSDFDEKIGLDKLEKLAYS-DPVMSDNYSG
NDUF2.2_CELE        RQFKTGG-----------TLGKLWMS--ERVSDFDDQIGLDKLEKLAYS-DPVLSDNYEG
NDUF2_HCON          RQFKNTS------------VGRIWAS--DHYEEFDKAIGLEKIEKLGIE-GPVWADNFEG
NDUF2_NAME          RQFKSCG-----------GMGKLWMS--EKYDDYDKAIGLDKLEKLTYE-QPVFSDSYEG

NDUF2.1_FHEP        -----------L----NFGPQHPAAHGVLRLIMQLDGETVERLDPHIGFLHRGTEKLIEH
NDUF2.2_FHEP        KIRKRQISCFLLENASSYGQFCHDEFCQFVVAINAKFSRYPSLIPTAAI-----------
NDUF2_SMED          -EKEIEVVNVQL----NFGPQHPAAHGVLRCVLTLDGETILKADPHIGLLHRATEKLIEY
NDUF2_SMAN          NVSHQEVQSVHL----NFGPQHPAAHGVLRLIMELDGEKIIRLDPHIGLLHRGTEKLIEY
NDUF2_HMIC          -VRHYEVQSIQL----NFGPQHPAAHGVLRMIMELDGEIIIRLDPHVGFLHRGTEKLMEY
NDUF2_EGRA          -FRHFEVQSLQL----NFGPQHPAAHGVLRLVMELDGEIIIRLDPHVGFLHRGTEKLMEY
NDUF2_EMUL          -FHHFEVQSLQL----NFGPQHPAAHGVLRLVMELDGEIVIRLDPHVGFLHRGTEKLMEY
NDUF2_TMUR          DLIERKVQNMML----NFGPQHPAAHGVLRLMLELDAEIVLRAIPHVGLLHRATEKLIEY
NDUF2.2_OVOL        ECNEKPLHYYLL----NFGPQHPAAHGVLRLVLELDNEMVMKAVPHVGLLHRGTEKLIEY
NDUF2.2_BMAL        DFHEKPLHYYLL----NFGPQHPAAHGVLRLILELDNEMVMKAVPHIGLLHRATEKLIEY
NDUF2.1_OVOL        EVHEKPLHYYLL----NFGPQHPAAHGVLRLVLELDNEMVMKATPHIGLLHRATEKLIEY
NDUF2.1_BMAL        EMHEKPLHYYLL----NFGPQHPAAHGVLRLILELDNEMVMKATPHIGLLHRGTEKLIEY
NDUF2_MHAP          YPREKRIENMIL----NFGPNHPAAHGVLRLILKLEGEVVIKAIPHIGLLHRATEKLIEY
NDUF2.2_ASUU        KLREKALENMIL----NFGPQHPAAHGVLRLVLKLEGEVIIKAMPHIGLLHRGTEKLMEH
NDUF2_SRAT          KKREKILENMIL----NFGPQHPAAHGVLRLVLKLEGEVIIKAIPHIGLLHRATEKLIEH
NDUF2.1_ASUU        RLREKSLENMVL----NFGPQHPAAHGVLRLVLKLEGEVIIKATPHIGLLHRGTEKLIEY
NDUF2.1_CELE        KQREKNLENMIL----NFGPQHPAAHGVLRLVLKLEGEVIIKAIPHIGLLHRATEKLIEH
NDUF2.2_CELE        KKREKNLENMIL----NFGPQHPAAHGVLRLVLKLEGEVIIKAIPHIGLLHRATEKLIEH
NDUF2_HCON          KRREKELENMIL----NFGPQHPAAHGVLRLVLKLEGEVIIKAIPHIGLLHRGTEKLIEH
NDUF2_NAME          KHREKQLENMIL----NFGPQHPAAHGVLRLVLKLEGEVIIKAIPHIGLLHRATEKLIEH
                               *    .:*      .  :   :  . .      *  .:

NDUF2.1_FHEP        KTYLQALPYFDRLDYVSPMAQEHAYSLCVEKLLGITVPPRAQYLRVIFVEITRILNHLLN
NDUF2.2_FHEP        --SSTALPYFDRLDYASMMCNEQGYCLAVEKLLNIEVPPRAKYIRTLFAELTRLLNHCLA
NDUF2_SMED          KTYTQALPYFDRLDYVSMMCNEQCYSLAVEKLLNINIPPRAKYIRTLFGELTRLLNHAMS
NDUF2_SMAN          KTYTQALPYFDRLDYASMMCNEQCYSLAVEKLLNIEVPPRAKYIRTLYSEITRIMNHCLA
NDUF2_HMIC          KTYTQNLPYVDRLDYFSMMCSEQCYSLAVEKLLGIDIPKRAKYIRTMFAEITRILNHCIA
NDUF2_EGRA          KTYTQNLPYVDRLDYFSMMSSEQCYSLAVEKLLGIEIPPRAKYIRTMFAEVTRLLNHCCA
NDUF2_EMUL          KTYTQNLPYVDRLDYFSMMSSEQCYSLAVEKLLGIEIPPRAKYIRTMFAEVTRLLNHCCA
NDUF2_TMUR          KTYTQALPYFDRLDYVSMMTNEEAFALAVEKLLGIDIPPRAKWIRVLFAELTRILNHILA
NDUF2.2_OVOL        KTYTQALPYFDRLDYITMLTNEQGFALAVEKLLGIEIPLRAKWIRTLFAELNRIANHTFS
NDUF2.2_BMAL        KTYTQALPYFDRLDYITMLTNEQGFALAVEKLLGIEIPPRAKWIRTLFAELNRLANHAFS
NDUF2.1_OVOL        KTYTQAVPYFDRMDYVSMMCNETAFALAVEKLLGVNIPPRAKFIRTLMNELTRIQNHLMG
NDUF2.1_BMAL        KTYTQAVPYFDRMDYVSMMCNETAFALAVEKLLGIDIPPRAKFIRTLMNELTRIQNHLMG
NDUF2_MHAP          KTYTQAMPYFDRLDYVSMMCNEQAWSLAVEKLLGIDIPPRAKWIRTLFAELTRIQNHIMG
NDUF2.2_ASUU        KTYTQCMPFMDRLDYVTMLCSEQAFALAIEKLLGIEVPPRAKWIRTLFAELNRIANHAFG
NDUF2_SRAT          KTYTQALPYFDRLDYVSMMCNEQAYSLAVEKLLGITPPPRAQMIRIFMAEMTRIQNHIMG
NDUF2.1_ASUU        KTYTQALPYFDRLDYISMMCNEGAFALAIEKLLRIDVPPRAKFIRTLVYELTRLQNHIMG
NDUF2.1_CELE        KTYTQALPYFDRLDYVSMMCNEQAWSLAVEKLLGIDIPTRAKYIRTLMGELTRIQNHIMG
NDUF2.2_CELE        KTYTQALPYFDRLDYVSMMCNEQAFSLAIEKLLGIDVPPRAKYIRILFGELTRIQNHIMG
```

```
NDUF2_HCON          KTYTQALPYFDRLDYVSMMCNEQGFALAVEKLLGIEIPPRAKWIRTLMAELTRIQNHVMG
NDUF2_NAME          KTYTQALPYFDRLDYVSMMCNEQGFALAVEKLLGIDIPPRAKYIRTLFAELTRIQNHIMG
                          :*:.**:** : : .*  :.*.:**** :  * **: :* :  *:.*: **

NDUF2.1_FHEP        VTTHALDVGAMNPLFWMFEEREKMLSFYEKASGARFHAAYIRPGGLAADIPDGLDEEIIS
NDUF2.2_FHEP        IGSHILDVGAITPIFWLFEEREKMFEFYERVSGARMHAAYFRPGGVYLDLPLGLMDDMYQ
NDUF2_SMED          IATHILDVGGLTPLFWLFEEREKMLEFYERVSGARMHAAYIRPGGVAQDMPLGLMDDIYE
NDUF2_SMAN          VGSTVLDIGGITPIFWLFEEREKMFEFYERVSGARMHAAYIRPGGVYLDMPLGLMDDIYQ
NDUF2_HMIC          LGANILDMGAITPFFWLLEEREKLMEMYERASGARFHAAFIRPGGVAIDIPLGFMEDLYE
NDUF2_EGRA          LGSNILDMGAITPFFWLFEEREKLMEMYERASGARFHAAYVRPGGVSLDIPLGFMEDLYG
NDUF2_EMUL          LGSNILDMGAITPFFWLFEEREKLMEMYERASGARFHAAYVRPGGVSLDIPLGFMEDLYG
NDUF2_TMUR          VSSHALDIGALTPLFWLFEEREKMLEFYERCSGARMHANYIRPGGVAVDIPIGLLDDMYD
NDUF2.2_OVOL        IVTHALDIGAMTPLFWMFEEREKLFEFNERVSGARMHVNYIRPGGVAYDLPLGLLDDIYD
NDUF2.2_BMAL        IVTHALDIGAMTPLFWMFEEREKLFEFNERVSGARMHVNYIRPGGVAWDLPLGLLDDIYD
NDUF2.1_OVOL        ITTHALDIGAMTPLFWMFEEREKMFEFTERVCGARMHSNYVRPGGVAWDLPLGWMDDVYD
NDUF2.1_BMAL        VTTHALDVGAMTPLFWMFEEREKMFEFTERACGARMHSNYVRPGGVAWDLPLGWMDDVYD
NDUF2_MHAP          ITTHALDVGALTPFFWMFEEREKLFEFSERVSGARMHANYIRPGGVAWDLPLGLLDDIYD
NDUF2.2_ASUU        VTTHALDIGAMTPLFWLFEEREKLFEFNERVSGARMHINYFRPGGVSYDLPLGLLDDIYD
NDUF2_SRAT          ITTHALDVGAMTPFFWMFEEREKMFEFAERVSGARMHVNYVRPGGVAWDLPLGYLDDVYD
NDUF2.1_ASUU        ITTHALDIGAMTPLFWMFEEREKLFEFTERISGARMHANYVRPGGVAWDLPLGWMDDVYD
NDUF2.1_CELE        ITTHALDVGAMTPFFWMFEEREKLFEFSERVSGARMHANYVRPGGVAWDLPIGLMDDIYD
NDUF2.2_CELE        ITTHALDVGAMTPFFWMFEEREKLFEFSERVSGARMHANYVRPGGVAWDLPVGLMDDIYD
NDUF2_HCON          ITTHALDIGAMTPFFWMFEEREKMFEFSERVSGARMHVNYIRPGGVAWDLPIGLMDDIYD
NDUF2_NAME          ITTHALDIGAMTPFFWMFEEREKMFEFSERVSGARMHVNYVRPGGVAWDLPLGLMDDIYD
                    : :  **:*.:.*:**::*****::.: *. .***:*  :.****:  *:* *  :::

NDUF2.1_FHEP        FLESFPHKLDDVADVLTDNPIFKQRLVDIGKVSKKEAVALGFSGPVLRASGIPWDLRRSQ
NDUF2.2_FHEP        FLEKFVQRIDELCDMTLTNPIWINRTRDVGIVSAEDALDMGFSGVMLRGSGIQWDLRKTQ
NDUF2_SMED          FLVKFPQRLEEVEDVVTHSPIWVARTKDIGILSAEDAINYGMSGPMLRGSGIRWDLRKAQ
NDUF2_SMAN          FIQKFPQRLDEISDLLFDNPIWITRTKDVGIVSAEDALDLGFSGVMLRGSGIKWDLRKTQ
NDUF2_HMIC          IIEKLPQRLDELEDLLCTNPLWIHRTKDIGVVSAEDAIDLGFSGVMLRGSGVQWDLRKTQ
NDUF2_EGRA          IIQKLPQRLDEVEDLLITNPLWIHRTKGIGVVSAEDAIDLGFTGVMLRGSGVQWDLRKTQ
NDUF2_EMUL          IIQKLPQRLDEVEDLLITNPLWIHRTKGIGVVSAEDAIDLGFTGVMLRGSGVQWDLRKTQ
NDUF2_TMUR          WLRKFPARLDELDDVLTESRIWKQRTKDIGVVTAENALNWGFSGVMLRGSGIKWDLRKTQ
NDUF2.2_OVOL        WAIQFPQRIDTMEDMLTENRIWKSRTLDIGLVSAADAQNLGFSGIMLRGSGVKYDVRKTA
NDUF2.2_BMAL        WTIQFPQRIDAMEDMLTENRLWQSRTRDIGLISATDAQSLGFSGVMLRASGVKYDVRKAQ
NDUF2.1_OVOL        WAIRFPQRLDMMEDLLTGNRIFLSRTVDIGLVRAEDALLWGFSGVMLRGSGIKWDIRKAQ
NDUF2.1_BMAL        WALRFPQRLDMMEDLLTGNRIFMSRTVDIGLVKAEDALLWGFSGVMLRGSGIKWDIRKAQ
NDUF2_MHAP          WSTKFPARIDELEDLLTDNRIWKKRLIDIGIVKAADALNWGFSGVMLRGSGIKWDLRKTQ
NDUF2.2_ASUU        WAEKFPERIDELEDMLTDNRIWKARTIDIGLVSAADALNFGYSGVMLRSSGVKWDVRKAT
NDUF2_SRAT          WCVKFPQRIDELEDMLTENRIWKQRTQDVGIVTASDALNWGFSGVMLRGSGIKWDVRKSS
NDUF2.1_ASUU        WAVKFPERIDELEDMLTENRIWKARTIDVGIVTASDALNWGFSGVMLRGSGIKWDVRKTQ
NDUF2.1_CELE        WAIKFPERIDELEDMLTENRIWKARTIDIGLVSAADALNWGFSGVMVRGSGIKQDVRKTE
NDUF2.2_CELE        WAVKFPARIDELEDMLTENRIWKARTVDIGLVSASDALNWGFSGVMVRGSGIKQDVRKTE
NDUF2_HCON          WAVKFPERIDELEDMLTENRIWKARTVDIGLVSAADALNWGFTGVMVRGSGIKQDVRKTQ
NDUF2_NAME          WAIKFPERIDELEDMLTENRIWKARTVDIGLVSASDALNWGFSGVMVRGSGIKQDVRKTQ
                        :  .:: : *:   . ::  *  .:* :   :*   * :* ::*.**:  *:*.:

NDUF2.1_FHEP        PYEVYESLDFAIPVGNCGDSYD----------RYLVRMAEMYESVKIIKQCIDRLPEGPV
NDUF2.2_FHEP        PYDAYADMEFDVPIGINGDCYD----------RFAVRLEEMRQSIRIMQQCLNKMPAGEV
NDUF2_SMED          PYDAYDEMEFDIPIGVNGDCYD----------RYLIRFEEMRQSIRIMLQCMNKMPPGEI
NDUF2_SMAN          PYDAYEDMDFDVPVGVHGDCYD----------RFIVRMEEMRQSLRIMEQCLNKMPKGEI
NDUF2_HMIC          PYDAYEDMEFDVPIGVNGDVYD----------RFIIRIEEVRQSCRIILQCLNKMPSGEI
NDUF2_EGRA          PYDAYEDMDFDVPIGVNGDVYD----------RFILRIEEMRQSCRIVLQCLNKMPPGEI
NDUF2_EMUL          PYDAYEDMDFDVPIGVNGDVYD----------RFILRIEEMRQSCRIVLQCLNKMPPGEI
NDUF2_TMUR          PYEVYDKVEFDVPVGTKGDCYD----------RYLIRMEEMRQSVRIMEQCLNKMPPGEV
NDUF2.2_OVOL        PYEVYDQIEFDVPVGTKGDCYD----------RYLCRLEEIRQSLRIILHCLNKMPSGEI
NDUF2.2_BMAL        PYEVYDQIEFDVPVGTRGDCYD----------RYLCRLEEMRQSLRIVLDCLNKMPPGEI
NDUF2.1_OVOL        PYDAYDQVDFDVPIGVKGDCYD----------RYLVRMEEMRESLKIIFECLNKMPPGEI
NDUF2.1_BMAL        PYDAYDQVDFDVPIGVKGDCYD----------RYLIRMEEMRESVRIIFECLNKMPAGEI
NDUF2_MHAP          PYDAYSEVEFDVPIGTKGDCYD----------RYLCRVMEMRESLKIILQCLNKMPRGEV
NDUF2.2_ASUU        PYDAYDQVEFDVPVGTKGDCYD----------RYLCRVEEMRQSLRIVHQCLNKMPAGEV
NDUF2_SRAT          PYDGYEKIDFDVPIGTRGDCYD----------RYLCRLEEMRQSLKIILHCLNNMPPGEI
```

```
NDUF2.1_ASUU       PYDAYEELEFDVPVGKNGDCYD----------RYLCRVEEMRQSLRLVLQCLNKMPPGEI
NDUF2.1_CELE       PYDAYADMEFDVPIGTKGDCYD----------RYLCRIEEMRQSLNIVHQCLNKMPAGEI
NDUF2.2_CELE       PYDAYADMEFDVPIGTKGDCYD----------RYLCRVEEMRQSLNIVHQCLNKMPTGEI
NDUF2_HCON         PYEVYDQVEFDVPIGTKGDCYD----------RYLCRVEEMRQSLNIIHQCLNKMPMGEV
NDUF2_NAME         PYEVYDQLEFDVPIGTKGDCYDRLLLPTDAYVRYLCRIEEMRQSLRIVHQCLNKMPAGEI
                   **: * .::* :*:*  ** **          *:  *. *: :* .::  *::.:* * :

NDUF2.1_FHEP       VVDDRKIAPPSRAEMKTSMEALIHHFKLYSEGYHVPEGETYFAVESPKGEFGVYIVSDGT
NDUF2.2_FHEP       KVDDAKITPPKRSEMKDSMEALIHHFKLFTEGYQVPPGATYTAIEAPKGEFGVYLVSDGT
NDUF2_SMED         KVDDHKIVPPSRSEMKDSMEALIHHFKLFTEGYQVPPGTTYTAIESPKGELGVYLVSDGS
NDUF2_SMAN         KVDDAKICPPKRAEMKSSMEALIHHFKLFSEGYLVPPGSTYTAVEAPKGEFGVYLVSDGT
NDUF2_HMIC         KIDDAKICPPTRAEMKNSMEALIHHFKLFTEGYSVPPGVTYTAVEAPKGEFGVYMVSDGT
NDUF2_EGRA         KVDDAKICPPKRAEMKSSMEALIHHFKLFTEGYSVPPGVTYTAVEAPKGEFGIYLVSDGS
NDUF2_EMUL         KVDDAKICPPKRAEMKSSMEALIHHFKLFTEGYSVPPGVTYTAVEAPKGEFGVYLVSDGS
NDUF2_TMUR         RVDDYKVVPPRRADMKKSMEALIHHFKFFSEGFQVPPGSTYTAIEAPKGEFGVYVVSDGS
NDUF2.2_OVOL       KVDDHKVVPPKRSEMKNTMESLIHHFKFFSEGYQVPPGSCYVPIEAPNGEYGTYLVADGT
NDUF2.2_BMAL       KVDDHKVVPPKREEMKNSMESLIHHFKFFSEGYQVPPGSCYVPIEAPNGEYGTYVVADGT
NDUF2.1_OVOL       KIDDHKVVPPKRAEMKQNMESLIHHFKYYTEGYQVPPGATYVPIEAPKGEFGVYLVSQGE
NDUF2.1_BMAL       KVDDHKVVPPKRAEMKQNMESLIHHFKYYTEGYQVPPGATYVPVEAPKGEFGVYLVSHGE
NDUF2_MHAP         KVDDQKIMPPSRAEMKESMEALIHHFKFFTEGFQVPPGHVYVPTEAPKGEFGVYLVADGT
NDUF2.2_ASUU       KVEDHKITPPKRGEMKTSMESLIHHFKFFTEGFQVPPGSCYVPIEAPNGEFGTYIVADGT
NDUF2_SRAT         KVDDNKVVPPKRAEMKESMESLIHHFKFFTEGYQVPPGATYVATEAPKGEFGVYLVADGT
NDUF2.1_ASUU       KVDDHKVVPPKRSEMKESMEALIHHFKFFTEGFQVPPGATYLPVEAPKGEFGVYLLADGT
NDUF2.1_CELE       KVDDHKVVPPKRAEMKENMESLIHHFKFFTEGFQVPPGATYVPIEAPKGEFGVYLVADGT
NDUF2.2_CELE       KSDDHKVVPPKRAEMKENMESLIHHFKFFTEGFQVPPGATYVPIEAPKGEFGVYLVADGT
NDUF2_HCON         KVDDHKIVPPKRAEMKDSMESLIHHFKFFTEGYQVPPGATYVPIEAPKGEFGVYLVADGT
NDUF2_NAME         KVDDHKVVPPKRAEMKDSMESLIHHFKFFTEGYQVPPGATYVPIEAPKGEFGVYLVADGT
                     :* *: ** * :** .**:****** ::**: ** *  * . *:*:** * *::: *

NDUF2.1_FHEP       NRPYRCRIRAPGFVHLQALDTLSRKHLLADVPAILGSLDIVFGEVDR
NDUF2.2_FHEP       NRPYRCKIKAPGFAHLAGIDKLCRGYLLADVVAVI----VISSNCDR
NDUF2_SMED         NRPYRCKIKAPGFVHLAALHKLSKGLMLADLVAIIGTLDVVFGEVDR
NDUF2_SMAN         NKPYRCKIKAPGFAHMAAIDKLCRGFMLPDVVAVIGTLDIVFGEVDR
NDUF2_HMIC         NIPYRVKLRAPSFAHLAASNKLCKGYLLADVVSVLGNLDLVFGEVDR
NDUF2_EGRA         NIPYRCKFRTPSFPHLAACNKLCKGFLLADVVSVLGNLDIVFGEVDR
NDUF2_EMUL         NIPYRCKFRTPSFPHLAACNKLCKGFLLADVVSVLGNLDIVFGEVDR
NDUF2_TMUR         SKPYRCYIRAPGFAHLSKIEAVSKYHFLADLVAIIGTMDIVFGEVDR
NDUF2.2_OVOL       SKPYRCFIRGPGFAHLAGLPHLTYMSMISDVVAVIGTLDIVFGEVDR
NDUF2.2_BMAL       SKPYRCFIRGPGFAHLAGLHDLTHMSLISDVVAVIGTMDIVFGEVDR
NDUF2.1_OVOL       SRPYRCFARSPGFPHLAAIDDICHMSLLSDVVAVIGTLDLVFGEIDR
NDUF2.1_BMAL       SKPYRCFARSPGFPHLAAIDDICHLSMLSDVVAVIGTLDLVFGEIDR
NDUF2_MHAP         SKPYRCYIRAPGFAHLAKIHDISYMHMIPDLVAIIGTLDVVFGEIDR
NDUF2.2_ASUU       SKPYRVFIRGPSFPHLASLPDVAYMSLIADVVAIIGTLDIVFGEVDR
NDUF2_SRAT         SKPYRCFIRAPGFAHLAALHDVTYMSLIADVVAVIGTMDIVFGEVDR
NDUF2.1_ASUU       TKPYRCFLRAPGFAHLSVIHDICYMSLIADMVAVIGTLDLVFGEVDR
NDUF2.1_CELE       GKPYRCFIRAPGFAHLAAIHDVCYMSLIADIVAVIGTMDIVFGEVDR
NDUF2.2_CELE       GKPYRCFIRAPGFAHLAAIHDVCYMSLIADIVAVIGTMDIVFGEVDR
NDUF2_HCON         SKPYRCYIRAPGFAHLAAMHDICYMALIADVVAVIGTLDIVFGEVDR
NDUF2_NAME         SKPYRCYIRAPGFPHLAAIHDICYMSLIADVVAVIGTLDIVFGEVDR
                     ***   . *.* *:     :    ::.*: :::    :: .: **
```

NDUF7

```
NDUF7.2_FHEP       ------------------------------------------------------------
NDUF7.1_TMUR       MLAKQLLGGSQMKAALSALLARSYCTN-------------------------KPLTDVKP
NDUF7.2_TMUR       ------------------------SSK-------------------------EPLEGLKP
NDUF7_OVOL         ALTSSSLEDYFILSIRLSIASVMNFISFVSGKIMQTTNSAGMFLNLRRVFATMTCQIRRE
NDUF7_BMAL         ------------------------------------------------------------
NDUF7_ASUU         MVVSCLHQQLFVTSTELVVSIHVPNIL----------------------------LAVQM
NDUF7_SRAT         --------------------------------------------------------TQIS
NDUF7_CELE         ------------------------------------------------------------
NDUF7_NAME         ------------------------------------------------------------
NDUF7_HCON         ------------------------------------------------------------
```

```
NDUF7.1_SMED       ------------------------------------------------------------
NDUF7.2_SMED       ------------------------------------------------------------
NDUF7_SMAN         ------------------------------------------------------------
NDUF7.1_FHEP       ------------------------------------------------------------
NDUF7_HMIC         -------------SALCAE-----------------------------------------
NDUF7_EGRA         MLSLARTARCVCVPKLCSPCTLYSSSK---------------------------------
NDUF7_EMUL         MLSLVRTARCVCVPKLCSPCTLYSSSK---------------------------------

NDUF7.2_FHEP       -------------------------------------------------VATLYNVVHL
NDUF7.1_TMUR       GDEVPVDPYGAVPAELDKPAK--------GV-PWTGSPFT-KTSNMAEYALARVDDIINW
NDUF7.2_TMUR       GDKLPLDPYGPVPAEADSPPK--------DI-SWTGSPFT-KTSNMAEYALARADDLINW
NDUF7_OVOL         SSKSIGPTSPMAPVEKGTRPK--------GIWGVIGSPWQ-DPLPKGEMAVTRLDDLNNL
NDUF7_BMAL         --------------------K--------GIWGIIGSPWQ-DPLPKGEMAVTRLDDLNNM
NDUF7_ASUU         TLEAPQHLIVEHLCWEENADKITYLWMFQGMWGKFGSPFT-GPMSTGEYALARVDDVINL
NDUF7_SRAT         QVATGSNIVKKIPTNHDEAPK--------GI-GKTGTPFL-ETKSLAEYALARVDDVINM
NDUF7_CELE         --------------SNSEAPK--------GI-ATTGTPFL-NPSSKAEYALARLDDVLNL
NDUF7_NAME         --------------EPGEAPK--------GM-ATTGTPFV-KTSSAGEYAMARIDDVMNM
NDUF7_HCON         -------------------PK--------GI-AKTGTPFL-DTSSKAEYALARLDDVMNM
NDUF7.1_SMED       ----------------QKTPK--------IVDDQEHSPLKGPFSNKVEIAVARLDDIINF
NDUF7.2_SMED       ----SQFSDGKSNEKTKTDAK--------LEEVTPNSPLQVPLTNKVEFALAKLDDLINF
NDUF7_SMAN         -----SSSEPSQPSKVDSSDE--------SIPFKPYSPFP-KPQGKIDYVITRLDDLADW
NDUF7.1_FHEP       -------------QNADLSPE--------PVKYKPYSPFQ-GTASKTEYVIARADDLINW
NDUF7_HMIC         ----PNSLKQPAPNPNKPYEE--------VEKYKPYSPFRVSPSNKAEYVVARMDDVVNW
NDUF7_EGRA         -STVPESLKQPAPKPDHPYEE--------VSKYKPYSPFRVTPSNKVEYALARLDDLINW
NDUF7_EMUL         -STVSESLKQPAPKPDHPYEE--------VSKYKPYSPFRVTPSNKVEYALARLDDLINW
                                                                     ::   ::

NDUF7.2_FHEP       ILSASNSLWPLTFGLACCAMEMMQMAGPRYDMDRFGVVFRASPRQADLMIVAGTVTNKMA
NDUF7.1_TMUR       --TRRGSLWPLSFGLACCAIEMMHFAAPRYDMDRFGVVFRASPRQADCIIVAGTVTNKMA
NDUF7.2_TMUR       --TRRGSLWPLSFGLACCAIEMMHFAAPRYDMDRFGVVFRASPRQADCMIVAGTVTNKMA
NDUF7_OVOL         --IHRCSLWPMTFGLACCAVEMMHFAAPRYDMDRFGVVFRASPRQVDVMIVAGTVTNKMA
NDUF7_BMAL         --IHRCSLWPMTFGLACCAVEMMHFAAPRYDMDRFGVVFRASPRQSDVMIVAGTVTNKMA
NDUF7_ASUU         --CQRTSLWPLTFGLACCAVEMMHFAAPRYDMDRYGVVFRASPRQVDLIFIAGTVTNKMA
NDUF7_SRAT         --CQRTSVWPLTFGLACCAVEMMHMAAPRYDMDRYGVVFRASPRQCDLIFVAGTVTNKMA
NDUF7_CELE         --AQRGSIWPLTFGLACCAVEMMHFAAPRYDMDRYGVVFRASPRQADLIFVAGTVTNKMA
NDUF7_NAME         --VQRTSLWPLTFGLACCAIEMMHFAAPRYDMDRYGVVFRASPRQTDLIFVAGTVTNKMA
NDUF7_HCON         --VQRTSLWPLTFGLACCAIEMMHFAAPRYDMDRYGVVFRASPRQTDLIFVAGTVTNKMA
NDUF7.1_SMED       --GRKGSIWPMTFGLACCAVEMMHMAAPRYDMDRFGILFRASPRQSDVIIVAGTLTNKMA
NDUF7.2_SMED       --GRKGSIWPMTFGLACCAIEMMHMAAPRYDMDRFGLLFRASPRQTDVIIVAGTLTNKMA
NDUF7_SMAN         --ARKNSIYPLTFGLACCAVEMMHIAGPRYDMDRYGVVFRASPRQADLLIVSGTVTNKMA
NDUF7.1_FHEP       --ARKNSLWPLTFGLACCAVEMMHIAGPRYDMDRFGVVFRASPRQSDVLIVAGTVTNKMA
NDUF7_HMIC         --ARKNSIWPISFGLACCAIEMMQMAGPRYDMDRFGVVFRASPRQTDLMIVAGTLTNKMA
NDUF7_EGRA         --GRKNSIWPVTFGLACCAIEMMHMAAPRYDMDRFGVVFRASPRHCDLMIVAGTLTNKMA
NDUF7_EMUL         --GRKNSIWPVTFGLACCAIEMMHMAAPRYDMDRFGVVFRASPRHCDLMIVAGTLTNKMA
                         *::*::*******:***::*.*******:*::******: * ::::**:*****

NDUF7.2_FHEP       PAFRRIWDQMTHPKWAISMGSCANSGGYYHYSYSVVRGADRIIPIDLYVPGS-----LFD
NDUF7.1_TMUR       PALRRIYDQMPDPKWVISMGSCANGGGYYHYAYSVVRGCDRLIPVDIYVPGCPPTAEALL
NDUF7.2_TMUR       PALRRIYDQMPNPKWVISMGSCANAGGYYAYSYSVVRGVDRIVPVDIYIPGCPPSAEALL
NDUF7_OVOL         PAVRRVYDQMPEPKWVISMGSCANGGGYYHYSYSVLKGVDRIIPVDIYVPGCPPSAEALL
NDUF7_BMAL         PAVRRVYDQMPEPKWVISMGSCANGGGYYHYSYSVLKGVDRIIPVDIYVPGCPPSAEALL
NDUF7_ASUU         PALRRIFDQMPEAKWVISMGSCANGGGYYHYAYSVLKGCDRVIPVDIYVPGCPPTAEALL
NDUF7_SRAT         PALRRIYDQMPEPKWVISMGSCANGGGYYHYAYSVLRGCDRVIPVDIYVPGCPPTAESLL
NDUF7_CELE         PALRRIYDQMPEAKWVISMGSCANGGGYYHYAYSVLRGCDRVIPVDIYVPGCPPTAEALL
NDUF7_NAME         PALRRVYDQMPEAKWVISMGSCANGGGYYHYAYSVLRGCDRVIPVDIYVPGCPPTAEALL
NDUF7_HCON         PALRRVYDQMPEAKWVISMGSCANGGGYYHYSYSVLRGCDRVIPVDIYVPGCPPTAEALL
NDUF7.1_SMED       TALRKIYDQMHYPKWVISMGSCANGGGYYHYSYSVVRGCDRIIPVDIYVPGCPPSAEALL
NDUF7.2_SMED       TALRKVYDQMYPPKWVIAMGSCAIGGGYYHYSYSVVRGCDRIIPCDIFVPGCPPSAEALL
NDUF7_SMAN         PALHRIYSQMTQPKWVISMGSCANGGGYYHYSYSVVRGCDRIIPVDIYVPGCPPSAEALM
NDUF7.1_FHEP       PALRRIYDQMTHPKWAISMGSCANGGGYYHYSYSVLRGCDRLVPIDIYVPGCPPSAEALM
NDUF7_HMIC         PALRRIWDQMPHPKWAISMGSCANGGGYYHYSYSVVRGVDRIIPIDIYVPGCPPTAEALL
NDUF7_EGRA         PALRRIWDQMPHPKWAISMGSCANGGGYYHYSYSVVRGCDRIIPVDIFVPGCPPTAEALL
```

```
NDUF7_EMUL          PALRRIWDQMPHPKWAISMGSCANGGGYYHYSYSVVRGCDRIIPVDIFVPGCPPTAEALL
                    .*...::.**  .**.*:***** .**** *:***:.* **::* *:::**.      :

NDUF7.2_FHEP        FRLRDFCTKAEFALHQERWYRPIDLNWAGSRGHVSIHRRCLRRATFVEKRDVHASKIDMG
NDUF7.1_TMUR        YGVLQLQKKIKNSKRAQMWYRQ--------------------------------------
NDUF7.2_TMUR        YGVLQLQKKIKNSRRAQMWYRH--------------------------------------
NDUF7_OVOL          YGVLQLQKKIKRKREGLMWHRR--------------------------------------
NDUF7_BMAL          YGVLQLQKKIKRKRESLMWHRR--------------------------------------
NDUF7_ASUU          YGVLQLQKKIKRKREALLWYRR--------------------------------------
NDUF7_SRAT          YGVLQLQKKIKRKRNAQLWYRK--------------------------------------
NDUF7_CELE          YGVLQLQKKIKRKREAQLWYRR--------------------------------------
NDUF7_NAME          YGVLQLQKKIKRKREAQLWYRR--------------------------------------
NDUF7_HCON          YGVLQLQKKIKRKREAQLWYRR--------------------------------------
NDUF7.1_SMED        YGFLQLQKKIKRMDSSQMWYRK--------------------------------------
NDUF7.2_SMED        YGFLQLQKKIKRHNASQMWYRK--------------------------------------
NDUF7_SMAN          YSILQLQKKIKSEKHFKSWYRR--------------------------------------
NDUF7.1_FHEP        YAILQLQKRIKYMHTQYSWYRK--------------------------------------
NDUF7_HMIC          YSILQLQKKIKYMRTQQFWYRK--------------------------------------
NDUF7_EGRA          YSVLQLQKKIKYMKTQQMWYRK--------------------------------------
NDUF7_EMUL          YSVLQLQKKIKYMKTQQMWYRK--------------------------------------
                    : . :: .. :       *:*

NDUF7.2_FHEP        SA
NDUF7.1_TMUR        --
NDUF7.2_TMUR        --
NDUF7_OVOL          --
NDUF7_BMAL          --
NDUF7_ASUU          --
NDUF7_SRAT          --
NDUF7_CELE          --
NDUF7_NAME          --
NDUF7_HCON          --
NDUF7.1_SMED        --
NDUF7.2_SMED        --
NDUF7_SMAN          --
NDUF7.1_FHEP        --
NDUF7_HMIC          --
NDUF7_EGRA          --
NDUF7_EMUL          --
```

Supplementary Figure 1. Alignments of complex II and nuclear encoded quinone-binding complex I subunits. Multiple sequence alignments of SDHA, SDHB, SDHC, SDHD, NDUF-2 and NDUF-7. Alignment were performed in MUSCLE (v3.8). In grey are highlighted the conserved sequences identified by Gblocks 0.91b.
