# Supplementary material for: Complex I and II Subunit Gene Duplications Provide Increased Fitness to Worms
Source: Front Genet. 2019 Oct 25;10:1043. doi: 10.3389/fgene.2019.01043 (PMC6859908; doi:10.3389/fgene.2019.01043)
Supplement: Supplementary file 2 [file Image_2.pdf]

```
CLUSTAL O(1.2.1) multiple sequence alignment

SDHB

Ascaris      93ICGSCAMNIAGENTLACICNIDQNTSKTTKIYPLPHMFVIKDLVPDMNLFYAQYASIQPW
Celegans    677ICGSCAMNIGGQNTLACICKIDSDTSKSTKIYPLPHMFVVKDLVPDMNLFYAQYASIQPW
               *********.*:*******:**.:***:***********:********************

Ascaris     153LQKKTKINLGEKQQYQSIKEQEKLDGLYECILCACCSASCPSYWWNADKYLGPAVLMQAY
Celegans    737IQKKTPLTLGEKQMHQSVAERDRLDGLYECILCACCSTSCPSYWWNADKYLGPAVLMQAY
               :**** :.***** :**: *:::**************:**********************

Ascaris     213RWIIDSRDDSAAERLARMQDGFSAFKCHTIMNCTKTCPKHLNPARAIGEIKMLLTKMKTK
Celegans    797RWVIDSRDDYATERLHRMHDSFSAFKCHTIMNCTKTCPKHLNPAKAIGEIKSLLTGFTSK
               **:****** *:*** **:*.***********************:****** *** :.:*

SDHC

Ascaris      34EKTPIQVWGWDYLMRQRALKRPIAPHLTIYKPQMTWMVSGLHRVTGCAMAGTLLIGGVGF
Celegans    865AKTPIQKFGWEYLLKQRSKNRPIAPHLTVYQPQLTWMLSGFHRISGCVMAGTLLVGGIGF
                ***** :**:**::**: :********:*:**:***:**:**::**.******:**:**

Ascaris      94SVLPLDFTTFVEFIRGLGIPWVILDTFKFIIAFPIAFHTLNGIRFIGFDMAKGTD-IPSI
Celegans    925AVLPFDFTAFVDFIRSWNLPCAVTAVFKYIIAFPIIFHTLNGIRFLGFDLAKGVNNVGQI
               :***:***:**:***.  :* .:  .**:****** *********:***:***.: : .*

SDHD

AscHipo      88LALALTLHVHWGVWGVVNDYGRPFVLGDTLAAAVRVGAYIFTACLLAGLLYFNEHDVGLT
AscNorm    1069LTVAIVLHVHWGIAGVVSDYARPFVIGDTLARVARASVYIITVILLASLLHFNNSDVGLT
Celegans   1069LTVALTLHIHWGIHGVVYDYARPYVIGEAAAKAAHVGVYLITGLLLGALLHFNTNDVGIT
               *::*:.**:***: *** **.**:*:*:: * ..:...*::*  **..**:**  ***:*
```

Supplementary Figure 2. Sequence alignment of the complex II subunits involved in quinone binding. Colored residues correspond to those included in the docking search space. Color code: green = complete identity; yellow = strong identity; orange = weak identity; red = no identity.
