# Supplementary material for: Complex I and II Subunit Gene Duplications Provide Increased Fitness to Worms
Source: Front Genet. 2019 Oct 25;10:1043. doi: 10.3389/fgene.2019.01043 (PMC6859908; doi:10.3389/fgene.2019.01043)
Supplement: Supplementary file 3 [file Image_3.pdf]

NDUF2

```
                   206              419       451
NDUF2.1_FHEP       VTTHALDVGAMNPLFWM…VESPKGEFG…SRKHLLADVPAILGSLDIVFGEVDR
NDUF2.2_FHEP       IGSHILDVGAITPIFWL…IEAPKGEFG…CRGYLLADVVAVI----VISSNCDR
NDUF2_SMED         IATHILDVGGLTPLFWL…IESPKGELG…SKGLMLADLVAIIGTLDVVFGEVDR
NDUF2_SMAN         VGSTVLDIGGITPIFWL…VEAPKGEFG…CRGFMLPDVVAVIGTLDIVFGEVDR
NDUF2_HMIC         LGANILDMGAITPFFWL…VEAPKGEFG…CKGYLLADVVSVLGNLDLVFGEVDR
NDUF2_EGRA         LGSNILDMGAITPFFWL…VEAPKGEFG…CKGFLLADVVSVLGNLDIVFGEVDR
NDUF2_EMUL         LGSNILDMGAITPFFWL…VEAPKGEFG…CKGFLLADVVSVLGNLDIVFGEVDR
NDUF2_TMUR         VSSHALDIGALTPLFWL…IEAPKGEFG…SKYHFLADLVAIIGTMDIVFGEVDR
NDUF2.2_OVOL       IVTHALDIGAMTPLFWM…IEAPNGEYG…TYMSMISDVVAVIGTLDIVFGEVDR
NDUF2.2_BMAL       IVTHALDIGAMTPLFWM…IEAPNGEYG…THMSLISDVVAVIGTMDIVFGEVDR
NDUF2.1_OVOL       ITTHALDIGAMTPLFWM…IEAPKGEFG…CHMSLLSDVVAVIGTLDLVFGEIDR
NDUF2.1_BMAL       VTTHALDVGAMTPLFWM…VEAPKGEFG…CHLSMLSDVVAVIGTLDLVFGEIDR
NDUF2_MHAP         ITTHALDVGALTPFFWM…TEAPKGEFG…SYMHMIPDLVAIIGTLDVVFGEIDR
NDUF2.2_ASUU       VTTHALDIGAMTPLFWL…IEAPNGEFG…AYMSLIADVVAIIGTLDIVFGEVDR
NDUF2_SRAT         ITTHALDVGAMTPFFWM…TEAPKGEFG…TYMSLIADVVAVIGTMDIVFGEVDR
NDUF2.1_ASUU       ITTHALDIGAMTPLFWM…VEAPKGEFG…CYMSLIADMVAVIGTLDLVFGEVDR
NDUF2.1_CELE       ITTHALDVGAMTPFFWM…IEAPKGEFG…CYMSLIADIVAVIGTMDIVFGEVDR
NDUF2.2_CELE       ITTHALDVGAMTPFFWM…IEAPKGEFG…CYMSLIADIVAVIGTMDIVFGEVDR
NDUF2_HCON         ITTHALDIGAMTPFFWM…IEAPKGEFG…CYMALIADVVAVIGTLDIVFGEVDR
NDUF2_NAME         ITTHALDIGAMTPFFWM…IEAPKGEFG…CYMSLIADVVAVIGTLDIVFGEVDR
                   : :  **:*.:.*:**:  *:*:** *     ::.*: :::    :: .: **
```

NDUF7

```
                   64
NDUF7.2_FHEP       SLWPLTFGLACCAMEMMQMAG
NDUF7.1_TMUR       SLWPLSFGLACCAIEMMHFAA
NDUF7.2_TMUR       SLWPLSFGLACCAIEMMHFAA
NDUF7_OVOL         SLWPMTFGLACCAVEMMHFAA
NDUF7_BMAL         SLWPMTFGLACCAVEMMHFAA
NDUF7_ASUU         SLWPLTFGLACCAVEMMHFAA
NDUF7_SRAT         SVWPLTFGLACCAVEMMHMAA
NDUF7_CELE         SIWPLTFGLACCAVEMMHFAA
NDUF7_NAME         SLWPLTFGLACCAIEMMHFAA
NDUF7_HCON         SLWPLTFGLACCAIEMMHFAA
NDUF7.1_SMED       SIWPMTFGLACCAVEMMHMAA
NDUF7.2_SMED       SIWPMTFGLACCAIEMMHMAA
NDUF7_SMAN         SIYPLTFGLACCAVEMMHIAG
NDUF7.1_FHEP       SLWPLTFGLACCAVEMMHIAG
NDUF7_HMIC         SIWPISFGLACCAIEMMQMAG
NDUF7_EGRA         SIWPVTFGLACCAIEMMHMAA
NDUF7_EMUL         SIWPVTFGLACCAIEMMHMAA
                   *::*::*******:***::*.
```

Supplementary Figure 3. Alignment of NDUF2 and NDUF7 subunits. The residues that form the quinone-reaction chamber according to Degli Esposti (2015) are highlighted in black. These residues are not all identical in helminth sequences, yet highly conserved. In turquoise is highlighted an unusual K→Y substitution found in nematodes that would be related to RQ-binding according to Degli Esposti (2015). We found that most nematodes encode Y, but some encode H and *T. muris* retained a K. In plathyhelminths this residue is either K or R. The numeration corresponds to *C. elegans* NDUF2-1 and NDUF7 sequences. Reference: Degli Esposti, M. (2015). Genome Analysis of Structure-Function Relationships in Respiratory Complex I, an Ancient Bioenergetic Enzyme. *Genome Biol. Evol.* 8, 126–47. doi:10.1093/gbe/evv239.
