# Supplementary material for: Complex I and II Subunit Gene Duplications Provide Increased Fitness to Worms
Source: Front Genet. 2019 Oct 25;10:1043. doi: 10.3389/fgene.2019.01043 (PMC6859908; doi:10.3389/fgene.2019.01043)
Supplement: Supplementary file 5 [file Table_1.docx]

**Supplementary Table 1**

p values for the expression changes during dauer stages (ANOVA test).

|  | Stage compared | p value |
| --- | --- | --- |
| *sdha-1* | Entry - Dauer | 0.03 |
|  | Entry - Exit | 0.08 |
|  | Dauer - Exit | 0.22 |
| *sdha-2* | Entry - Dauer | 0.25 |
|  | Entry - Exit | 0.99 |
|  | Dauer - Exit | 0.25 |
| *nduf2-1* | Entry - Dauer | 0.11 |
|  | Entry - Exit | 0.18 |
|  | Dauer - Exit | 0.86 |
| *nduf2-2* | Entry - Dauer | 0.34 |
|  | Entry - Exit | 0.26 |
|  | Dauer - Exit | 0.62 |
